# NK4 Antagonizes Tbx1/10 to Promote Cardiac versus Pharyngeal Muscle Fate in the Ascidian Second Heart Field

**DOI:** 10.1371/journal.pbio.1001725

**Published:** 2013-12-03

**Authors:** Wei Wang, Florian Razy-Krajka, Eric Siu, Alexandra Ketcham, Lionel Christiaen

**Affiliations:** Department of Biology, New York University, New York, New York, United States of America; King's College London, United Kingdom

## Abstract

The heart and head muscles share common developmental origins and genetic underpinnings in vertebrates, including humans. Parts of the heart and cranio-facial musculature derive from common mesodermal progenitors that express *NKX2-5*, *ISL1*, and *TBX1*. This ontogenetic kinship is dramatically reflected in the DiGeorge/Cardio-Velo-Facial syndrome (DGS/CVFS), where mutations of *TBX1* cause malformations in the pharyngeal apparatus and cardiac outflow tract. Cardiac progenitors of the first heart field (FHF) do not require *TBX1* and segregate precociously from common progenitors of the second heart field (SHF) and pharyngeal muscles. However, the cellular and molecular mechanisms that govern heart versus pharyngeal muscle specification within this lineage remain elusive. Here, we harness the simplicity of the ascidian larva to show that, following asymmetric cell division of common progenitors, *NK4/NKX2-5* promotes *GATAa/GATA4/5/6* expression and cardiac specification in the second heart precursors by antagonizing Tbx1/10-mediated inhibition of *GATAa* and activation of *Collier/Olf/EBF* (*COE*), the determinant of atrial siphon muscle (ASM) specification. Our results uncover essential regulatory connections between the conserved cardio-pharyngeal factor Tbx1/10 and muscle determinant *COE*, as well as a mutual antagonism between NK4 and Tbx1/10 activities upstream of *GATAa* and *COE*. The latter cross-antagonism underlies a fundamental heart versus pharyngeal muscle fate choice that occurs in a conserved lineage of cardio-pharyngeal progenitors. We propose that this basic ontogenetic motif underlies cardiac and pharyngeal muscle development and evolution in chordates.

## Abstract 2

Cross inhibition between NK4 and TBX1 transcription factors specifies heart versus pharyngeal muscle fates by promoting the activation of tissue-specific regulators in distinct precursors within the cardiopharyngeal lineage of the ascidian, *Ciona intestinalis*.

## Author Summary

Mutations in the regulatory genes encoding the transcription factors *NKX2-5* and *TBX1*, which govern heart and head muscle development, cause prevalent congenital defects. Recent studies using vertebrate models have shown that the heart and pharyngeal head muscle cells derive from common progenitors in the early embryo. To better understand the genetic mechanisms by which these progenitors select one of the two developmental trajectories, we studied the activity of these transcription factors in a simple invertebrate chordate model, the sea squirt *Ciona intestinalis*. We show that the sea squirt homolog of *NKX2-5* promotes early heart specification by inhibiting the formation of pharyngeal muscles. Conversely, the TBX1 homolog determines pharyngeal muscle fate by inhibiting GATAa and thereby the heart program it instructs, as well as promoting the pharyngeal muscle program through activation of *COE* (*Collier/Olf-1/EBF*), a recently identified regulator of skeletal muscle differentiation. Finally, we show that the NKX2-5 homolog protein directly binds to the *COE* gene to repress its activity. Notably, these antagonistic interactions occur in heart and pharyngeal precursors immediately following the division of their pluripotent mother cells, thus contributing to their respective fate choice. These mechanistic insights into the process of early heart versus head muscle specification in this simple chordate provide the grounds for establishing the etiology of human congenital cardio-craniofacial defects.

## Introduction

The combined cardiac and craniofacial malformations observed in Cardio-Velo-Facial/Di George Syndrome patients arise from complex sets of defects in the development of the pharyngeal apparatus [1]–[4]. Developmental studies focused on amniote model systems have documented the ontogenetic proximity of the branchiomeric pharyngeal muscles and the derivatives of the second heart field, which do express *TBX1/Tbx1*, *ISL1/Islet-1*, and *NKX2-5/Nkx2.5* [5]–[7]. Retrospective clonal analyses in the mouse established the lineage relationships within the anterior splanchnic mesoderm that gives birth to branchiomeric muscles and cardiac tissue (herein referred to as the cardio-pharyngeal mesoderm) [8],[9]. These studies demonstrated that cardiac progenitors of the first and second heart fields derive from common cardio-pharyngeal progenitors that also give birth to branchiomeric muscles, which are more closely related to second heart field precursors. In the mouse, this specific clonal motif is deployed independently in two parts of the anterior second heart field giving birth to the right ventricle and first arch muscles, on one hand, and to the outflow tract and second arch muscles, on the other hand [8],[10],[11]. Retrospective and prospective lineage studies combined with genetics and molecular analyses have illuminated developmental trajectories in the amniote pharyngeal mesoderm, but the relative complexity of vertebrate embryos has hindered the identification of progenitor cells and the analysis of the precise cellular characteristics of the heart versus pharyngeal muscles fate choice.

**Figure 1:**
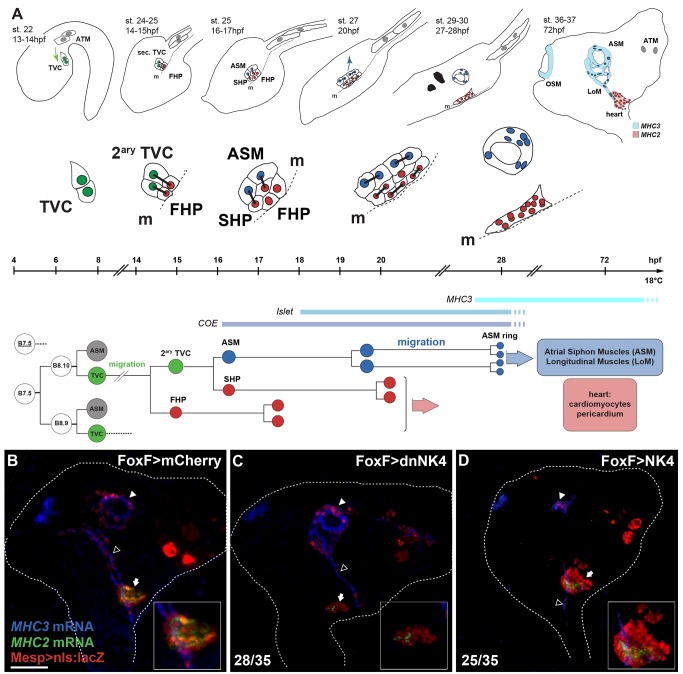
*NK4* promotes heart formation at the expense of the ASMs and LoMs in *Ciona intestinalis*. (A) Summary of early cardio-pharyngeal development: cell divisions, lineage, and migration of the TVCs. Green, TVCs and secondary TVCs (2^ary^ TVC); red, FHPs and SHP; blue, ASMs and LoM precursors; OSMs, Oral Siphon Muscles; ATMs, anterior tail muscles; hpf, hours postfertilization. Green and blue arrows, TVC and ASM migrations, respectively. (B–D) 72 hpf juveniles electroporated with Mesp>nls:lacZ (red) to mark B7.5 lineage cells and FoxF>mCherry (B), FoxF>dnNK4 (C), or FoxF>NK4 (D). Double FISH-IHC with *MHC2* (green) and *MHC3* (blue) probes mark cardiomyocytes and ASM/LoMs, respectively. White dotted lines outline whole bodies. Insets show magnified hearts. Numbers indicate juveniles showing the phenotype and total. Scale bar, 25 µm.

Tunicates are the closest living relatives of the vertebrates [12] and the ascidian *Ciona intestinalis* recently emerged as a simple and relevant model for chordate heart development [13],[14]. In ascidian embryos, each one of the bilateral B7.5 blastomeres uniquely expresses the conserved cardiac determinant *Mesp* and gives birth to four cells: two heart progenitors, the trunk ventral cells (TVCs), activate *FoxF* and *GATAa* and migrate into the trunk in response to an FGF signal, while their two sister cells form anterior tail muscles (ATMs) [15]–[20]. Electroporation of fertilized eggs with constructs using the *Mesp* or *FoxF* enhancers allows the visualization and molecular manipulation of B7.5 lineage cells or TVCs, respectively [16],[20]. Following migration, each bilateral pair of TVCs divides asymmetrically and medio-laterally to form small medial first heart precursors (FHPs) and large lateral secondary TVCs. The latter divide again asymmetrically to form small median second heart precursors (SHPs) and large lateral atrial siphon muscles (ASMs) precursors (Figure 1A) [21]. ASM-specific expression of the Collier/Olf-1/Ebf (COE) transcription factor triggers ASM specification, up-regulates the conserved LIM-homeobox gene *Islet*, and contributes to inhibiting the heart program specifically in the ASMs (Figure 1A) [21]. ASM precursors subsequently migrate dorsally and form a ring of cells expressing *myosin heavy chain 3* (*MHC3*) in the atrial siphon primordium. During metamorphosis, ASM precursors continue to divide and give birth to definitive ASM and longitudinal muscle (LoM) precursors, which migrate into the body wall [21],[22]. Meanwhile, the heart precursors divide and form a compartment containing *MHC2+* cardiomyocytes (Figure 1A,B). Here, we uncover regulatory mechanisms that distinguish the *COE*-expressing ASM from the SHPs following asymmetric cell divisions of the common TVC progenitors, thus providing insights into the cellular and molecular basis for heart versus pharyngeal muscle fate choice within the cardiogenic lineage.

## Results

### NK4 Activity Promotes Heart Specification at the Expense of ASMs

NK4 is the sole *Ciona* homolog of the conserved homeobox cardiac determinants *NK4/Nkx2-5*/*Csx/tinman* (Figure S1) [23],[24]. From the neurula to tailbud stages, *NK4* is broadly expressed in the ventral epidermis, anterior trunk endoderm, and TVCs, where its expression is regulated by Mesp, Fibroblast Growth Factor (FGF) signaling, FoxF, GATAa, and Bone Morphogenetic Protein (BMP) signaling (Figure S2) [15],[17],[19],[20],[24]–[26]. Fluorescent *in situ* hybridization and immunohistochemical (FISH-IHC) assays on Mesp>nls:lacZ-expressing larvae indicated that *NK4* expression persists at low levels until 20 hpf in both the ASMs, FHPs, and SHPs (Figure S2A–D). Expression became undetectable in the heart region during metamorphosis and at juvenile stages (Figure S2E–F′).

To investigate the TVC-specific functions of NK4, we generated a dominant-negative N448K DNA-binding domain mutant (dnNK4) [27],[28] and expressed dnNK4 or wild-type NK4 using the *FoxF* minimal TVC enhancer [20],[21]. We raised larvae electroporated with Mesp>nls:lacZ and either FoxF>mCherry, -NK4, or -dnNK4 to the early juvenile I stage (stage 38) [29] and performed double FISH-IHC using an anti–β-galactosidase antibody together with *MHC2* and *MHC3* antisense RNA probes to visualize the B7.5 lineage-derived cardiomyocytes and ASM/LoM, respectively (Figure 1A,B) [21]. In control juvenile hearts, *MHC2* marks differentiated β-Gal+ cardiomyocytes surrounded by β-Gal+/*MHC2−* cells, while *MHC3* is specifically expressed in the B7.5 lineage-derived, βgal+, ASMs, and LoMs and also in the A7.6 lineage-derived, βgal-, oral siphon muscles (OSMs) (Figure 1A,B) [21],[30]. Targeted expression of dnNK4 reduced the volume of the heart by ∼50% (903±92 µm^3^ per half versus 1,800±487 µm^3^ in controls; Table S1) and markedly inhibited *MHC2* expression in 80% of the juveniles (28/35; Figure 1C). In these animals, the ASM/LoM populations did not differ notably from control juveniles. Conversely, NK4 overexpression inhibited the formation of β-Gal+/*MHC3+* ASMs and LoMs (25/35; Figure 1D), while increasing the volume the TVC-derived β-Gal+ heart-like tissue by ∼250% (4,464±277 µm^3^ per half versus 1,800±172 µm^3^ in controls; Table S1). This indicates that sustained NK4 activity promotes heart specification at the expense of the ASM/LoM fate within the cardiogenic mesoderm.

### NK4 Activity Inhibits *COE* Expression in the SHPs

**Figure 2:**
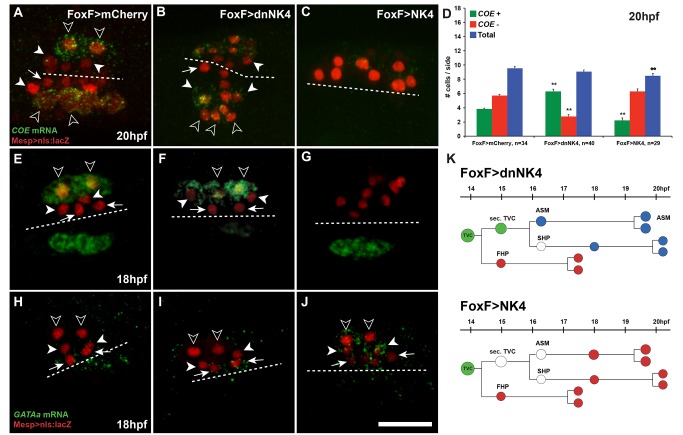
*NK4* represses *COE* and activates *GATAa* in TVC derivatives. (A–C, E–G, H–J) Larvae electroporated with Mesp>nls:lacZ (red), FoxF>mCherry (A, E, H), FoxF>dnNK4 (B, F, I), and FoxF>NK4 (C, G, H). FISH of *COE* transcripts (green) at 20 hpf (A–C) and 18 hpf (E–G) and *GATAa* transcripts (green) at 18 hpf (H–J). White dotted lines indicate the midline. Target expression of –dnNK4 in TVC lineage induces ectopic *COE* expression in SHPs (white arrowheads), in addition to ASM precursors (open arrowheads), but not in the FHPs (B, F, arrows). Conversely, FoxF>NK4 represses *COE* expression (C, G). In wild-type embryos, endogenous *GATAa* expression is restricted to heart precursors (arrows, H). FoxF>dnNK4 does not affect *GATAa* expression in the FHP (arrows, I). In FoxF>NK4 expressing larvae, *GATAa* transcripts can be detected in all TVC derivatives, including the lateral-most (open arrowheads). Scale bar, 25 µm. (D) Numbers of βGal and *COE* expressing cells per electroporated half at 20 hpf, in mCherry controls, dnNK4, or *NK4* expressing larvae. *n*, number of embryo halves scored. Error bar, standard error of the mean (SEM), Student's *t* test compared experimental condition to the control. **p<0.05. (K) Interpretation of TVC lineage fate re-programming by Fox>dnNK4 and FoxF>NK4. FoxF>dnNK4, SHPs are converted into ASMs; FoxF>NK4, ASMs are converted into heart precursors.

Since the ASM determinant *COE* is expressed immediately after the asymmetric division of the secondary TVCs (Figures 1A and S3) [21], we investigated the effects of NK4 activity on early *COE* expression. The majority (82%, 28/34) of control 20 hpf larvae contained four βgal+/COE+ and six βgal+/COE− cells per electroporated half (averaging 3.8±0.01 *COE+* and 5.7±0.17 *COE*− cells per half, *n* = 34; Figure 2A,D). Importantly, this pattern can be interpreted clonally given that stereotyped cell divisions separate the *COE*− heart and *COE+* ASM precursors (Figure 1A). Targeted expression of dnNK4 increased the number of *COE+* cells to 6.3±0.31, at the expense of *COE*− cells (2.8±0.25 per half, *n* = 40; Figure 2B,D). The total number of βgal+ TVC-derived cells in dnNK4-expressing larvae did not significantly change by 20 hpf, indicating that *COE* was ectopically expressed in cells that do not normally activate it (Figure 2D). Conversely, NK4 overexpression abolished *COE* expression in 24% (7/29) of electroporated larvae. Mosaic incorporation of the transgenes reduced the number of *COE+* cells to 1–3 per half in 38% (16/29) of the larvae. The average number of *COE+* cells was reduced to 2.2±0.4 (*n* = 29), and the number of βgal+, *COE*− cells increased to 6.3±0.4 (*n* = 29, Figure 2C,D). Targeted NK4 and dnNK4 expression also exerted opposite negative and positive effects on *COE* expression, respectively, in 18, 24, and 28 hpf larvae (Figures 2E–G and S4A–I). Notably, since TVC-specific expression of dnNK4 caused additional cells to express *COE* and migrate towards the atrial siphon placode in 24 and 28 hpf larvae, the absence of a clear excess of ASM/LoM cells in “dnNK4-derived” juveniles suggests that a reduction in ASM/LoM proliferation counteracts the effects of an initial excess of precursors on the final ASM/LoM population. By contrast, the heart did not seem to compensate for either the excess or reduction of the initial number of precursors since both dnNK4 and NK4 affected the juvenile heart volumes. Taken together, these results indicate that sustained NK4 activity inhibits *COE* expression in the TVC derivatives, which could explain its negative effects on ASM/LoM development.

To test if *NK4* regulates heart versus ASM fate specification by inhibiting *COE* expression, we performed epistasis assays by analyzing ASM ring formation and *MHC3* expression in 28 hpf larvae following combined inhibitions of NK4 and COE functions (Figure S5). In control 28 hpf larvae, 7.25±0.1 ASM cells per side formed typical rings containing 5.5±0.2 *MHC3+* cells (*n* = 43; Figure S5A,G,H). Note that we recently identified an unsuspected diversity among ASM precursors, whereby only a fraction of the cells expressed *MHC3* and began to differentiate before metamorphosis (Razy-Krajka et al., under review), which is consistent with the above observation. Targeted dnNK4 expression increased the number of ASM cells to 8.5±2.8, including 6.6±0.4 *MHC3+* cells (*n* = 35; Figure S5D,G,H). Co-expression of a dominant negative form of COE lacking the N-terminal DNA binding domain (COEΔ321) [31] reduced the excess ASM migration and ectopic *MHC3* expression induced by dnNK4 (Figure S5C,F–H). The constitutive repressor COE:WRPW blocked ASM migration and *MHC3* expression regardless of NK4 activity (Figure S5B,E,G,H). These data indicate that ectopic activation of *COE* mediates the ASM-promoting effects of dnNK4, further supporting the notion that NK4-mediated inhibition of *COE* expression is required to promote heart versus ASM fate specification.

The *Ciona* ortholog of *Islet1* (*Ci-Islet*) is up-regulated by COE in the ASM [21]. FISH-IHC indicated that *Islet* is expressed in all TVC daughter cells, but more highly in the ASMs compared to the heart precursors (Figure S3) [21]. We previously identified a COE-dependent ASM-specific *Islet* enhancer, which suggested that ASM-specific transcriptional inputs downstream of COE govern *Islet* expression [21]. Here, using intron-specific antisense RNA probes, we detected nascent *Islet* transcripts in all the TVC derivatives starting around 18 hpf, after the two asymmetric divisions, but exclusively in the ASM in 20 hpf larvae (Figure S3). These data show a widespread pan-TVC, but transient, activation followed by ASM-specific maintenance, thus emphasizing the complexity of *Islet* regulation in the cardio-pharyngeal mesoderm. Consistent with a predominant maintenance of *Islet* downstream of COE in the ASM, targeted expression of dnNK4 or NK4 caused up-regulation or reduction of *Islet* expression, respectively (Figure S6). Thus, as it is the case in mice [32] and zebrafish [33], NK4 activity appears to negatively regulate *Islet* expression in the cardiogenic mesoderm, at least in part through its effects on *COE*.

### NK4 Activity Promotes *GATAa* Expression in Secondary TVC Derivatives

To test whether NK4 overexpression converted the ASM into heart precursors, we assayed expression of the conserved early heart marker *GATAa*, which is highly expressed in the TVC progenitors at early tailbud stages but appears to be down-regulated by the late tailbud stage, before the first asymmetric division [17]. *GATAa* became undetectable in either secondary TVCs or FHPs after the first asymmetric division (Figure S7B). Expression became detectable again around 18 hpf, primarily in the FHPs, and appeared weaker in the SHPs (Figure S7C). We used *GATAa* intron-specific antisense RNA probes to detect nascent transcripts every half-hour after the end of TVC migration and further clarify the dynamics of *GATAa* transcription in the TVC derivatives. Two nuclear dots of nascent transcripts were detected in each one of the TVCs towards the end of their migration (Figure S7E). Following the first asymmetric cell divisions, nascent transcripts were undetectable in the FHPs and rarely in the secondary TVCs (Figure S7F,G). Notably, after the asymmetric division of secondary TVCs in 16 hpf larvae, *GATAa* nascent transcripts became detectable again in the FHPs but not in the SHP until 18.5 hpf, when nascent *GATAa* transcripts were still observed in the FHPs and became detectable in the SHPs but not in the ASM (Figure S7H–L). These observations illuminate the dynamics of *GATAa* transcription in the heart precursors: *GATAa* first shuts off following TVC migration before being reactivated successively in the FHPs and only about 2.5 h later in the SHPs.

We next examined *GATAa* expression following manipulations of NK4 activity. *GATAa* expression in the ∼18 hpf FHPs did not significantly change upon targeted expression of dnNK4, while the possibility of a reduced SHP expression could hardly be evaluated due to low expression levels in control animals (Figures 2I, S4, and S7). These data suggest that reactivation of *GATAa* in the FHPs is independent of NK4 activity. In contrast, we observed that overexpression of NK4 up-regulated SHP expression of *GATAa* and caused ectopic activation in the lateral-most TVCs at 18 hpf (Figures 2J and S4). Therefore, NK4 activity appears sufficient to promote cardiac-specific *GATAa* expression in the secondary TVC derivatives, and it may be necessary for *GATAa* reactivation specifically in the SHPs.

The above observations point to different requirements for NK4 activity in the FHPs and SHPs. TVC-specific expression of dnNK4 never converted all of the derivatives into ASM/LoM (Figure 1B; Table S1), nor did we observe ectopic activation of *COE* in all Mesp>nls:lacZ positive TVC derivatives (Figures 2B,D and S4). DnNK4 caused ectopic *COE* expression as early as 18 hpf, in cells positioned nearest to the ASM precursors (Figures 2B,F and S4). This stereotyped cell distribution suggested that only SHPs activated *COE* in response to dnNK4, while the *GATAa+* FHPs failed to activate *COE* upon mis-expression of dnNK4 (Figure 2K). Using intron-specific probes, we detected ectopic transcription of *COE* in the nuclei closer to the ASM, as early as 17–17.5 hpf but not between 15.5 and 16.5 hpf (Figure S4K). We hypothesized that only the derivatives of the secondary TVCs are competent to activate *COE* and become ASM precursors, while an NK4-dependent mechanism blocks *COE* activation in the SHPs, which then reactivate *GATAa* expression with a ∼2.5 h delay compared to the FHPs and form heart tissue.

### NK4 Activity Restricts *Tbx1/10* Expression to the ASM Precursors

**Figure 3:**
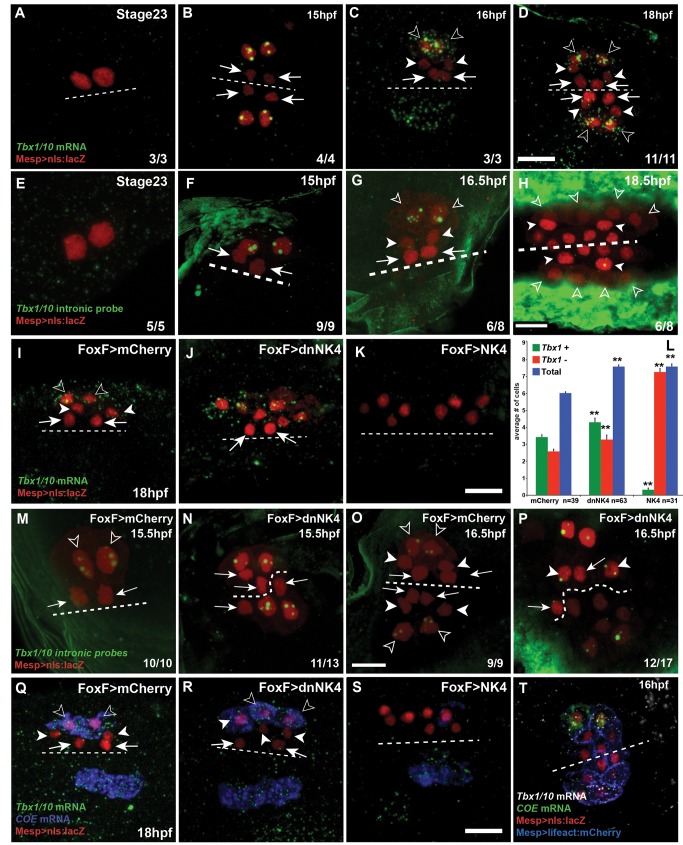
NK4 contributes to restricting *Tbx1/10* expression to the ASM precursors. (A–D, I–K and Q–S) *Tbx1/10* mRNAs (green), (E–H, M–P) *Tbx1/10* nascent transcripts (green) and (Q–S) *COE* mRNA (blue). Larvae electroporated with Mesp>nls:lacZ (red), FoxF>mCherry (I, M and O), FoxF>dnNK4 (J, N and P), and FoxF>NK4 (K). Ventral views, dotted lines indicate the midline; hpf, hours postfertilization; stage 23, 14 hfp at 16°C. Scale bar, 10 µm. Targeted expression of dnNK4 in the TVCs induces ectopic *Tbx1/10* expression in the SHP, but not in the FHPs (J arrows). FoxF>NK4 represses *Tbx1/10* expression in all TVC derivates (K). (L) Histograms showing the number of *Tbx1+* and βGal+ cells per half. *n*, number of embryo halves scored. Student's *t* test compared experimental condition to the control. **p<0.05, Error bar, standard error of the mean (SEM). *COE* transcripts can only be detected in lateral *Tbx1/10* positive cells in control larvae at 18 hpf (Q). –dnNK4 causes expansion of *COE* expression to the medial SHPs, which express *Tbx1/10* (R). Both *Tbx1/10* and *COE* expressions were inhibited by NK4 overexpression (S). (T) Larva (16 hpf) co-electroporated with Mesp>nls:lacZ (red, nuclei) and Mesp>LifeAct:mCherry (blue, cell cortex) and hybridized with *COE* (green) and *Tbx1/10* (white) probes. *Tbx1/10* transcripts were detected in both ASM and SHPs, but not in the FHPs.

In vertebrate development, the T-box DNA binding transcription factor TBX1 is an essential determinant of the pharyngeal mesoderm expressed in both the second heart field and branchiomeric muscle progenitors [3],[34],[35]. We previously reported that the *Ciona* homolog *Tbx1/10* is expressed in the LoM precursors in metamorphosing juveniles [21]. Here, using FISH-IHC, we found that *Tbx1/10* expression is initiated specifically in the large lateral secondary TVCs, after the first asymmetric division (Figure 3A,B). Following the second asymmetric division, high *Tbx1/10* expression is maintained in the ASM precursors at 16 to 18 hpf, while gradually fading in the SHPs (Figure 3C,D). Using *Tbx1/10* intron-specific probes, we confirmed the secondary TVC-restricted activation of *Tbx1/10* expression and found that the SHPs cease to transcribe *Tbx1/10* shortly after the second asymmetric division (Figure 3G), while nascent transcripts could still be detected in the ASM precursors (Figure 3E–H).

Double FISH-IHC showed that *COE* and *Tbx1/10* are co-expressed in the ASMs, while the SHPs retain only low amounts of *Tbx1/10* transcripts in 16 hpf larvae (Figure 3Q,T). Thus, the common progenitors of the ASM and SHPs specifically express *Tbx1/10*, a conserved essential regulator of pharyngeal mesoderm development, which is maintained specifically in the ASM precursors and rapidly shut off in the SHPs.

We first asked whether NK4 could be responsible for the SHP-specific termination of *Tbx1/10* expression. DnNK4 overexpression in the TVC progenitors significantly increased the numbers of *Tbx1/10*+ cells, from 3.4±0.1 cells per side in control larvae to 4.3±0.2 (*p* = 0.0027, Figure 3E,F,K). We confirmed these data using intron-specific probes that showed active *Tbx1/10* transcription in the SHPs of FoxF>dnNK4-expressing, but not in control 16.5 hpf larvae (Figure 3M–P). Notably, *Tbx1/10* expression did not expand to the FHPs in response to dnNK4, indicating that the restriction of *Tbx1/10* activation to the secondary TVCs is independent of NK4 activity (Figure 3J,N,P). Conversely, NK4 overexpression completely suppressed TVC-specific expression of *Tbx1/10* in 74% (23/31) of the larvae, thus reducing the average number of *Tbx1/10+* cells to 0.3±0.1 per side (Figure 3K,L). Taken together, these data indicate that NK4 activity is required to inhibit *Tbx1/10* expression in the SHPs.

Since targeted expression of dnNK4 causes ectopic activation of *COE* at 18 hpf (Figures 2F and S4), we performed double FISH assays to test whether *Tbx1/10* and *COE* co-localize in control and experimental larvae. In control larvae, we observed *COE* transcripts in the lateral-most cells that display the highest levels of *Tbx1/10* expression (Figure 3Q,T). In dnNK4-expressing larvae, ectopic *COE* only expanded to the medial SHP cells that maintained high *Tbx1/10* expression (Figure 3R). Finally, both *Tbx1/10* and *COE* expression were strongly inhibited by NK4 overexpression (Figure 3S). These observations open the possibility that Tbx1/10 defines the competence of the secondary TVC derivatives to activate *COE* and form ASM/LoM precursors.

### NK4 Antagonizes Tbx1/10-Mediated *GATAa* Inhibition and *COE* Activation

**Figure 4:**
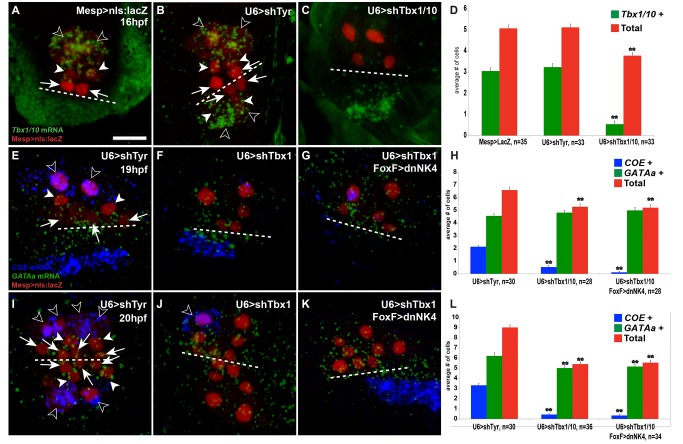
RNAi-mediated loss of Tbx1/10 function inhibits *COE* and causes ectopic *GATAa* expression. Open arrowheads, ASM precursors; white arrowheads, SHPs; arrows, FHPs. Dotted lines indicate the midline. (A–C) Larva (16 hpf) electroportated with Mesp>nls:lacZ (loading control) (A), U6>shTyr (shRNA targeting the pigment cell-specific *Tyrosinase*, used as negative control, B), and U6>shTbx1/10 (C). TVCs are labeled with Mesp>nls:lacZ (red) by immunostaining. U6>shTbx1/10 knocked down the endogenous *Tbx1/10* transcripts and induced a delay in secondary TVC division. Double FISH detection of *COE* (blue) and *GATAa* (green) expression in larva of 19 hpf (E–G) and 20 hpf (I–K). ShRNA-mediated knock-down of Tbx1/10 caused ectopic activation of *GATAa* in the lateral-most TVCs, while *COE* expression was markedly down-regulated (F, J). Note the persistent expression of *COE* in an ASM that did not receive the plasmids (open arrowhead in J). The co-electroporation of FoxF>dnNK4 with U6>shTbx1/10 shows no ectopic *COE* expression, but a remarkable down-regulation in the lateral-most TVCs. Ectopic *GATAa* expression is detected in the lateral-most TVCs as well as in larvae electroporated with U6>shTbx1/10 only (G, K). Histograms showing the number of *Tbx1/10*+, *COE*+, *GATAa*+, and βGal+ cells per half (D, H, and L). *n*, number of embryo halves scored. Student's *t* test compared experimental condition to the control. **p<0.05; error bar, standard error of the mean (SEM).

We first used short hairpin RNA (shRNA)-mediated RNA interference (RNAi) to knock down Tbx1/10 activity and test whether it is required to promote *COE* and inhibit *GATAa* expression in the cardio-pharyngeal mesoderm. We applied a plasmid-based strategy using RNA Polymerase III–mediated expression of shRNAs from a U6 promoter [36], which we slightly modified such that the hairpin structures mimick that of the endogenous *Ciona intestinalis* microRNA miR-2213 [37],[38]. We used a combination of shRNA constructs targeting two sites in the *Tbx1/10* coding region and that efficiently down-regulated expression of a GFP::Tbx1/10 fusion protein (unpublished data). Electroporation of U6>shTbx1/10 constructs down-regulated the endogenous *Tbx1/10* mRNA as evaluated by FISH (Figure 4A–D). Notably, U6>shTbx1/10 constructs induced a delay in secondary TVC division, which precluded the analysis of *COE* and *GATAa* expression at early time points (i.e., 16 to 18 hpf). In 19 and 20 hpf larvae, TVC divisions had resumed and double FISH-IHC assays using *COE* and *GATAa* probes showed that Tbx1/10 knock-down caused ectopic activation of *GATAa* in the lateral-most TVC derivative, while *COE* expression was markedly down-regulated (Figure 4E–L). These results indicate that Tbx1/10 activity is required to inhibit *GATAa* and promote *COE* expression in the ASMs.

We further tested whether the ectopic *COE* activation observed upon dnNK4 expression depends upon Tbx1/10 activity by co-electroporating FoxF>dnNK4 and U6>shTbx1/10 constructs and assaying *COE* and *GATAa* expression in 19 and 20 hpf larvae by double FISH-IHC (Figure 4G,H,K,L). These larvae were indistinguishable from larvae electroporated with U6>shTbx1/10 constructs alone, indicating that Tbx1/10 activity is required to mediate dnNK4-induced activation of *COE* in the SHPs, while NK4 activity appears dispensable to cause ectopic *GATAa* expression in the absence of Tbx1/10. These data also support the hypothesis that the remaining Tbx1/10 activity in the 15.5 to 17.5 hpf SHPs contributes to delaying *GATAa* reactivation. The latter eventually occurs probably because NK4 activity inhibits the maintenance of *Tbx1/10* expression.

**Figure 5:**
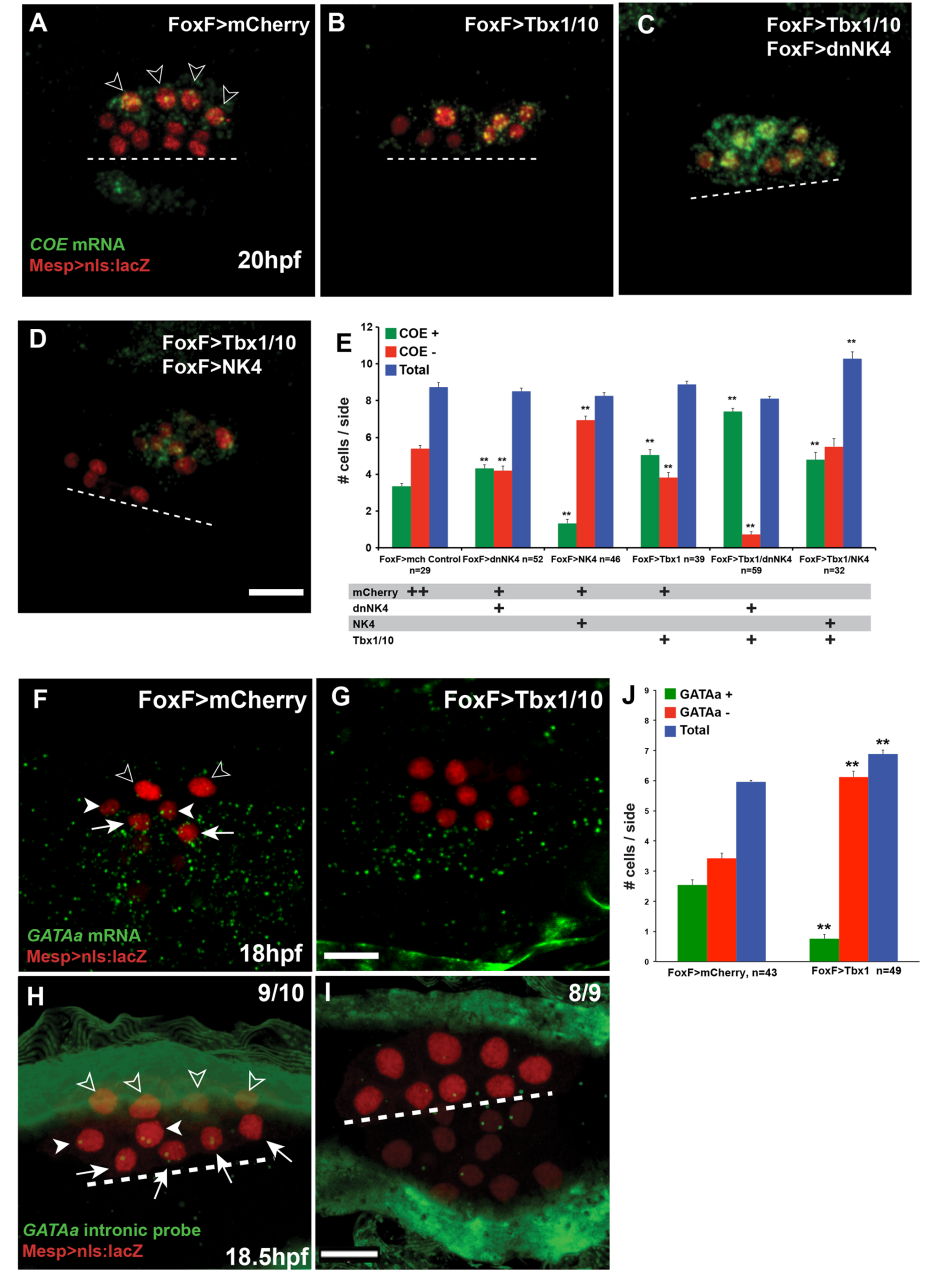
Tbx1/10 activity promotes *COE* and inhibits *GATAa* expression in the secondary TVC derivatives. (A–D, F–I) Larva electroporated with Mesp>nls:lacZ (red) and indicated combinations of FoxF>mCherry, FoxF>dnNK4, FoxF>NK4, and FoxF>Tbx1/10. FISH detection of *COE* mRNAs (green) at 20 hpf (A–D) and *GATAa* mRNAs (green) at 18 hpf (F, G) and *GATAa* nascent transcripts at 18.5 hpf (H, I). White dotted lines indicate the midline. ASM precursors, open arrowheads; SHPs, white arrowheads,;FHPs, arrows. Scale bar, 10 µm. In control larvae (FoxF>mCherry, A), *COE* expression is restricted to ASM precursors; with FoxF>Tbx1/10 (B), weak *COE* expression expanded to a subset of heart precursors. Combined FoxF>Tbx1/10 and FoxF>dnNK4 strongly activated *COE* expression in all TVC derivatives (C). FoxF>Tbx1/10 and FoxF>NK4 caused sporadic ectopic activation of *COE* (D). (E) Histograms showing the numbers of *COE+* and βGal+ cells per electroporated half. *n*, number of embryo halves scored. Student's *t* test compared experimental conditions to the control. **p<0.05; error bar, standard error of the mean (SEM). In the control, endogenous *GATAa* expression is restricted to heart precursors (F, H). FoxF>Tbx1/10 inhibits *GATAa* expression in all heart precursors (G, I). Histogram (J), numbers of βGal and *GATAa* expressing cells per electroporated half at 18 hpf. Error bar, standard error of the mean (SEM); Student's *t* test compared experimental condition to the control. **p<0.05.

We reasoned that the absence of *Tbx1/10* could explain why the FHPs expressed *GATAa* but did not activate *COE* and form ASMs in response to dnNK4 overexpression. We tested this possibility by mis-expressing Tbx1/10 and assaying *COE* expression at 20 hpf (Figure 5A–E). Tbx1/10 overexpression caused 56% (22/39) of the larvae to express *COE* in five or more cells per half (Figure 5B,E), resulting in a significant increase in the average number of *COE+* cells from 3.3±0.1 in controls (*n* = 29) to 5.1±0.3 in FoxF>Tbx1/10-electroporated larvae (*n* = 39, *t* test *p* = 8.2×10^−6^; Figure 5E). This ectopic expression of *COE* was similar to that observed with FoxF>dnNK4 (4.3±0.2, *n* = 52; Figures 2B,F and S4). However, upon targeted expression of either Tbx1/10 or dnNK4, three or four β-gal+ cells remained *COE*− in more than 50% of the electroporated halves (average numbers of *COE*− cells were 3.8±0.3 and 4.2±0.2 with Tbx1/10 and dnNK4, respectively). These cells likely represent the FHPs, suggesting that Tbx1/10 or dnNK4 alone are not sufficient to promote *COE* expression in the FHPs.

As reported above, *GATAa* is reactivated in the FHPs, where its expression appeared independent of NK4 activity, while the delayed reactivation in the SHPs may require NK4 function. We reasoned that persisting, NK4-sensitive, Tbx1/10 activity could be antagonizing *GATAa* expression specifically in the SHPs, but not in the FHPs that do not express *Tbx1/10*. We tested this possibility by mis-expressing Tbx1/10 in all TVC progenitors and assayed *GATAa* expression at 18 hpf. Tbx1/10 mis-expression eliminated all TVC-specific expression of *GATAa* in 57% (28/49) of the larvae, causing the average number of *GATAa+* cells to significantly decrease from 2.5±0.2 to 0.75±0.15 cells per side (*t* test *p* = 3.3×10^−11^; Figure 5F–J). Intron-specific probes indicated that Tbx1/10 mis-expression inhibited *GATAa* transcription in 18.5 hpf larvae (Figure 5I). Thus, Tbx1/10 is sufficient to inhibit *GATAa* reactivation in all TVC derivatives. These data, taken together with the ectopic *GATAa* observed upon *Tbx1/10* loss of function, indicate that remaining Tbx1/10 activity in the SHP may delay the reactivation of *GATAa* but that NK4 activity in the SHPs ultimately antagonizes Tbx1/10-mediated inhibition of *GATAa* reactivation, thus promoting heart fate specification in the SHPs.

To test whether NK4 could be inhibiting Tbx1/10-mediated activation of *COE* in addition to its inhibitory effect on the maintenance of *Tbx1/10* expression, we co-electroporated FoxF>Tbx1/10 and FoxF>dnNK4 and assayed *COE* expression at 20 hpf. This combination caused 68% (40/59) of the larvae to express *COE* in all TVC derivatives (Figure 5C,E). The number of *COE+* cells significantly increased to 7.4±0.2 (*n* = 59, *t* test *p* = 8.4×10^−31^; Figure 5C,E); at the expense of the β-gal+/*COE*− second heart precursors (SHPs) and FHPs (0.7±0.2 cells per half, *n* = 59; Figure 5C,E), while the total cell number did not change (8.1±0.1 versus 8.7±0.3 in control larvae). This shows that combining Tbx1/10 mis-expression and NK4 inhibition is sufficient to activate *COE* throughout the TVC progeny. Thus, in addition to its inhibitory effect upstream of *Tbx1/10* expression, NK4 activity is also normally required to prevent Tbx1/10-mediated activation of *COE* specifically in the SHPs.

Combined overexpression of Tbx1/10 and NK4 further tested the mutual Tbx1/10 versus NK4 antagonism upstream of *COE* expression. NK4 overexpression completely inhibited *COE* in >40% of the larvae, reducing the average number of *COE+* cells to 1.3±0.2 cells per half (*n* = 46; Figure 3E). Co-expression of Tbx1/10 rescued *COE*-expression to an average of 4.8±0.4 cells per half (*n* = 32; Figure 5D,E). These data indicate that NK4 exerts its inhibitory effects on *COE* expression both upstream and downstream of Tbx1/10 activity.

**Figure 6:**
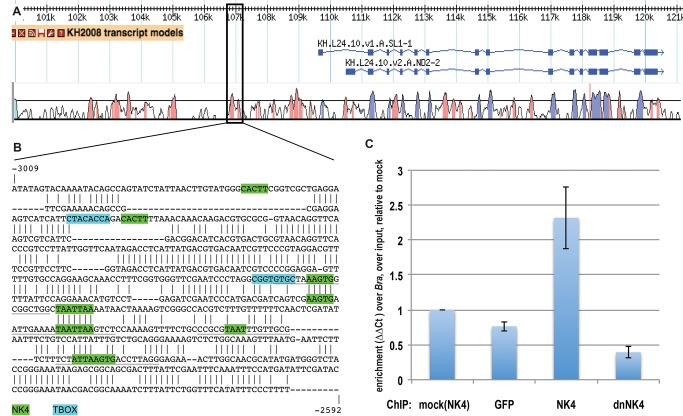
NK4 binds to the minimal ASM enhancer of *COE*. (A) Combined snapshots of the ANISEED [74] and VISTA [75] browsers showing the KH2008 transcript models [76] for *COE* and conservation between *Ciona intestinalis* and *Ciona savignyi*. Pink peaks indicate conserved noncoding sequences (>65% identity per 80 bp). (B) Alignment of the conserved noncoding region corresponding to the minimal ASM enhancer (Figure S8). Above sequence, *C. intestinalis*; bottom sequence, *C. savignyi*. Putative NK4 and Tbx1/10 sites are highlighted in green and blue, respectively. Coordinates are expressed relative to the translation start site (ATG, where A is +1). (C) ChIP-qPCR data expressed as average fold enrichment relative to the mock control (ChIP using nonspecific IgG on the chromatin extracted from larvae electroporated with FoxF>NK4:2xFLAG), for ChIP using an anti-FLAG tag antibody on chromatin samples obtained from larvae electroporated with GFP:2xFLAG, NK4:2xFLAG, or dnNK4:2xFLAG. Error bars, standard error of the mean (SEM), calculated over biological triplicates and qPCR performed with two primer pairs for *COE* and two pairs for the internal loading control *Brachyury*.

To test whether NK4 could directly repress *COE* expression, we isolated a minimal ASM enhancer located ∼2.5 kbp upstream of the translation start site (Figures 6 and S8). This *COE* minimal ASM enhancer overlaps with a noncoding sequence conserved between the sibling species *Ciona intestinalis* and *C. savignyi* (Figure 6A,B). Analysis of these sequences with the commercial Matinspector software [39] identified putative binding sites for NKX family transcription factors (Figure 6B). The algorithm did not detect putative TBX binding sites, but visual inspection identified two motifs similar to the conserved TBX half-site TCACACCT [40]–[42], as well as additional sequences resembling the high-affinity NKX2-5 binding site consensus TNAAGTG [43]. Because the possible TBX sites were not conserved between the two species, here we focused on testing whether NK4 binds directly to the *COE* enhancer. To this aim, we expressed FLAG-tagged versions of GFP, NK4, or dnNK4 using the FoxF minimal TVC enhancer and used chromatin immunoprecipitation followed by quantitative PCR (ChIP-qPCR) to evaluate NK4 binding to the endogenous *COE* enhancer. We used the *Brachyury* enhancer, which is only active in the notochord [44], as a negative control genomic region to normalize the qPCR data in each sample. The normalized *COE* enhancer enrichment over input was 2.3±0.1-, 3.1±0.1-, and 5.9±0.1-fold greater in the NK4-FLAG ChIP samples compared to mock, GFP-FLAG, and dnNK4-FLAG control samples, respectively (*p* = 0.036, *p* = 0.017, and *p* = 0.009, Table S2). These data support the hypothesis that NK4 directly represses *COE* transcription by binding to its minimal ASM enhancer and confirm that the N448K mutant is unable to bind NK4 cognate DNA.

**Figure 7:**
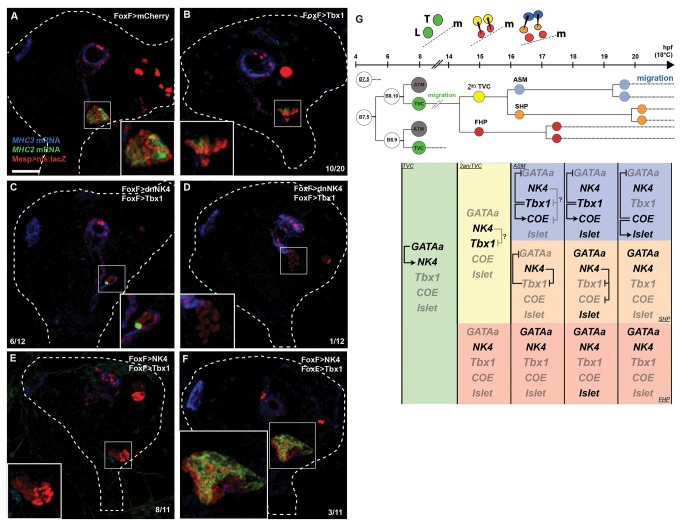
Combined Tbx1/10 mis-expression and NK4 inhibition can convert all TVC derivatives into differentiated ASMs and LoMs in 72 hpf juveniles. (A–F) Juveniles electroporated with Mesp>nls:lacZ(red) and indicated constructs were hybridized with *MHC3* (blue) and *MHC2* (green) probes. (A) FoxF>mCherry control. (B) FoxF>Tbx1/10 reduced heart volume and the number of *MHC2*+ cells in 10/20 animals; (C–D) combined FoxF>dnNK4 and FoxF>Tbx1/10 converted most (C) or all (D) TVC derivatives into differentiated ASMs and LoMs; (E–F) combined FoxF>NK4 and FoxF>Tbx1/10 showed least dramatic effects than each one alone: ASM/LoM formation defects are observed, 8/11 juveniles have reduced hearts and *MHC2* expression, and 3/11 juveniles have enlarged heart and increased *MHC2* expression. White dotted lines outline whole bodies. Insets show magnified hearts. Scale bar, 25 µm. (G) Summary model of the NK4 and Tbx1/10 mutual antagonism regulating cardiac versus ASM fate specification within a conserved clonal topology for progressive fate choices. The cells and approximate time windows are showed (i.e., align with the timeline above). Greyed labels indicate inactive genes and regulatory interactions. The question marks point to the unknown mechanism(s) that prevent(s) NK4-mediated inhibition of *Tbx1/10* and *COE* expression in the secondary TVCs and ASM founder cells.

Finally, we investigated whether manipulations of the NK4-Tbx1/10 cross-antagonism would affect heart and ASM/LoM differentiation after metamorphosis. To determine whether combining Tbx1/10 mis-expression and NK4 inhibition is sufficient to convert TVC derivatives into differentiated ASMs and LoMs, we raised larvae through metamorphosis and assayed *MHC2* and *MHC3* expression. Overexpression of Tbx1/10 alone reduced the average heart volume by ∼37% (1,131±128 µm^3^ per half, *n* = 9, versus 1,800±172 µm^3^, *n* = 8, in controls; Table S1) and markedly reduced *MHC2* expression in 50% of the juveniles (*n* = 10/20; Figure 7B). This effect is similar to that of dnNK4 (Figure 1B, Table S1), suggesting Tbx1/10 overexpression also converted the SHPs into ASM/LoM precursors. Combined expression of Tbx1/10 and dnNK4 further reduced the average heart volume by ∼77% (598±136 µm^3^ per half, *n* = 12 versus 1,800±172 µm^3^, *n* = 8, in controls; Table S1) and severely inhibited *MHC2* expression, with a corresponding increased development of *MHC3+* ASMs and LoMs (*n* = 6/12; Figure 7C,D). These results indicate that combining Tbx1/10 overexpression with NK4 inhibition can convert the TVC derivatives into ASM/LoM precursors, at the expense of the cardiac fate, in both the FHPs and SHPs.

Combined expression of Tbx1 and NK4 rescued the volume of heart-like β-gal+ tissue to 108% of controls, albeit reducing *MHC2* expression and causing conspicuous ASM/LoM defects (Figure 7E, Table S1). Conversely, 3/11 animals had an enlarged *MHC2+* heart, but did not entirely lack *MHC3+* ASM/LoMs (Figure 7F, Table S1). Taken together, these results support the notion that NK4 and Tbx1/10 can antagonize each other's effects upon heart versus ASM fate specification.

## Discussion

In summary, here we presented evidence that the precise spatio-temporal deployment of an NK4-Tbx1/10 antagonism governs heart versus ASM/LoM fate specification upstream of *COE* and *GATAa* in the ascidian cardio-pharyngeal mesoderm. We propose a model whereby the TVCs first undergo stereotyped asymmetric divisions that separate the lateral secondary TVCs and medial FHPs (Figure 7G). The lateral secondary TVCs activate *Tbx1/10* before undergoing another stereotyped asymmetric medio-lateral division. The large lateral ASM precursors activate *COE* in response to Tbx1/10 activity. In the SHPs, NK4 promotes cardiac specification by inhibiting *Tbx1/10* expression and antagonizing Tbx1/10-mediated inhibition of *GATAa* reactivation, activation of *COE* expression, and subsequent ASM development. Our preliminary ChIP-qPCR data indicate that NK4-mediated inhibition of *COE* expression in the SHPs might occur through direct repression. In the absence of *Tbx1/10* expression, the FHPs are the first to reactivate *GATAa* expression and assume a cardiac fate, possibly independently of NK4 activity.

A key aspect of the proposed model is the deployment of cross-regulatory interactions specifically in the secondary TVCs, SHPs, and/or ASMs. Even though the prevalent use of the minimal *FoxF* enhancer, which is active early in the TVCs [20], can cause precocious effects, several lines of evidence suggest that the proposed regulatory interactions do occur in the secondary TVCs and/or derivatives. First, the wild-type temporal profiles obtained using intron-specific probes showed that *Tbx1/10* transcription is quickly repressed specifically in the SHPs, while *GATAa* is reactivated with an approximate 2.5 h delay compared to the FHPs. Second, upon expression of dnNK4, *Tbx1/10* transcription was maintained specifically in the SHPs but did not expand to the FHPs, ectopic *COE* expression was also restricted to the SHPs, and *GATAa* expression in the FHPs was unaffected. We thus reasoned that our manipulations altered essential regulatory events that occur after the separation of the FHPs from the secondary TVCs and contributed to re-program specifically the SHPs into ASMs.

Our model also raises several questions that should be addressed in future studies. First, the above argument does not formally rule out the possibility that early TVC inputs (e.g., BMP signaling, see below and [26]) contribute to later *Tbx1/10*, *GATAa*, and/or *COE* expression in the secondary TVCs, FHPs, SHPs, and ASMs and could be affected by precocious manipulation of NK4 activity. Future studies capitalizing on enhancer analyses and whole genome profiling data (Razy-Krajka et al., under review) will uncover additional regulators of ASM versus heart fate specification. Second, since *NK4* transcripts were detected throughout the TVC lineage, and high levels of NK4 activity were sufficient to repress *Tbx1/10* and *COE* expression, what prevents NK4-mediated inhibition of *Tbx1/10* and *COE* expression in the secondary TVCs and ASM? Since differential regulatory inputs between the heart and ASM precursors occur following stereotyped oriented asymmetric divisions, we reason that differences between the SHPs and ASM arise as consequences of the asymmetric secondary TVC division. This may contribute to the observed delay in ectopic *COE* expression in the FoxF>dnNK4-expressing larvae, even though these cells maintained *Tbx1/10* transcription. Future studies ought to identify the molecular mechanisms that link oriented asymmetrical TVC division to the secondary TVC and ASM-specific inhibition of NK4-mediated repression of *Tbx1/10* and *COE*.

Previous studies of the ascidian heart progenitors indicated that early GATAa activity and BMP signaling control the TVC expression of *NK4*, which eventually feeds back negatively on two regulators of BMP activity, *Bmp2/4* and *Tolloid* [17],[26]. Regulatory interactions between BMP signaling, *NK4*, and *GATAa* orthologs are thought to be part of an ancient gene regulatory subnetwork referred to as the “heart kernel” [45],[46]. Activation of *pannier/GATA4/5/6* downstream of *tinman/NKX2-5* is one of the essential core regulatory interactions, which ascidians seemed to have rewired since the expression of *GATAa* starts before and determines that of *NK4* [17]. Our results indicate that, in turn, NK4 activity is required for *GATAa* reactivation in the SHPs but probably not in the FHPs. In addition, we propose that this interaction involves a double-negative gate network motif whereby NK4 activity prevents the maintenance of *Tbx1/10* expression, which would inhibit and actually contributes to delaying *GATAa* reactivation in the SHPs.

In mouse models for the Cardio-Velo-Facial/Di George syndrome, defects in the development of second heart field derivatives, such as the outflow tract, arise from altered Tbx1 dosage, including overexpression [47],[48]. Transcription profiling of the posterior pharyngeal mesoderm in *Tbx1* mutants and transgenic mice with additional copies of the human *TBX1* indicated that Tbx1 inhibits the expression of early heart differentiation markers, including *Tbx5* and *GATA4* [49]. Thus, the negative influence of Tbx1/10 activity on *GATA4/5/6* expression in the pharyngeal mesoderm is likely to be a conserved regulatory interaction in chordates. The inhibitory effects of Tbx1 on cardiac differentiation have been interpreted as a role for Tbx1 in maintaining a pluripotent progenitor state presumably required for proliferation of cardiac progenitors in the second heart field [34],[50]–[52]. In our study, secondary TVC divisions were markedly delayed in *Tbx1/10* knock-down experiments. Future studies will be required to determine how Tbx1/10 influences cell division and whether this delay indirectly contributes to the observed de-regulation of *COE* and *GATAa* in addition to the direct effect of the loss of Tbx1/10 function. Our observations suggest that ancestral roles of Tbx1/10 in the cardio-pharyngeal mesoderm were to promote cell divisions and oppose cardiac specification during pharyngeal muscle development.

What is the molecular mechanism for the proposed NK4-Tbx1/10 cross-antagonism? Our ChIP-qPCR data suggest that NK4 directly binds to and represses the *COE* ASM enhancer. The presence of putative TBX sites in this enhancer suggests that Tbx1/10 could also directly activate *COE*, which remains to be tested experimentally, as is the possibility of a direct repression of *Tbx1/10* expression by NK4. A strikingly similar regulatory mechanism was recently demonstrated for the mouse *Fgf10* enhancer, where Tbx1 binds and directly activates expression in the anterior second heart field, while Nkx2-5 also binds but represses enhancer activity in the myocardium, in part by competing with Islet1 for the same homeodomain binding sites [53]. Such a competition with Islet could also account for NK4-mediated repression of *COE* in the SHP, but presumably not at the initial stage of its expression since *Islet* is only activated about 2 h after *COE* in the TVC derivatives. Future studies will be required to determine if COE and Islet maintain each other's expression in the ASM precursors.

The NK4-Tbx1/10 antagonism possibly occurring at the *COE* enhancer might also involve direct protein–protein interaction. For instance, co-immunoprecipitation assays using tagged proteins indicated that Tbx1 and Nkx2-5 can physically interact in transfected 293T cells [54]. Interestingly, in this study Tbx1 and Nkx2-5 appeared to directly cooperate to activate the *Pitx2* ASE enhancer on the left side of the SHF [54]. Nkx2-5 was also shown to form a ternary complex with Tbx2 and the *Atrial natriuretic factor* (*Anf*) atrioventricular canal (AVC)-specific enhancer to directly repress the latter in the mouse myocardium [55]. Taken together, these and our data suggest that ancient molecular interactions between cardio-pharyngeal members of TBX and NKX families of transcription factors govern a range of enhancer activities underlying fate specification and differentiation. Future studies are required to determine the full extent of NK4 and Tbx1/10 binding using whole genome binding assays, investigate whether direct protein–protein interactions condition binding and enhancer activity, and finally identify potential novel regulators that may compete or cooperate with NK4 and Tbx1/10 in defining heart versus ASM identities.

Studies in vertebrates and flies have documented a requirement for Tbx1 activity in noncardiac muscle development. The *Drosophila* ortholog of *Tbx1/10*, *org-1*, contributes to myogenesis in the visceral mesoderm [56],[57]. In amniotes, the branchiomeric muscles derive from the pharyngeal mesoderm and are severely affected by impaired Tbx1 function, which is required for proper activation of the myogenic factors MyoD and Myf5 [3],[58]–[61]. A recent study identified the *COE* orthologs, *Ebf2* and *−3*, as essential determinants of MyoD expression and head muscle development in *Xenopus* [31]. Mis-expression of the cardio-pharyngeal determinant Mesp1 up-regulates *Ebf2* expression in mouse embryonic stem cells [62]. Taken together, these and our observations lead us to predict that Tbx1/10 genes act as conserved upstream regulators of *COE* orthologs during pharyngeal muscle development in vertebrates and tunicates.

Established lineage topologies within the mouse cardio-pharyngeal mesoderm show that precursors of the FHF diverge before the separation of the SHF from pharyngeal/branchiomeric muscle progenitors [8],[9]. *Nkx2.5* mutant mice display profound defects in the formation of SHF derivatives, which fail to proliferate, but form FHF-derived left ventricle tissue, and loss of Nkx2.5 function also up-regulates *Islet1* expression in the mouse cardiogenic mesoderm [32]. In zebrafish, SHF progenitors also reside in the *gata4+/nkx2.5+* part of the anterior lateral plate mesoderm and fail to proliferate normally in nkx2.5 morphant embryos, while initial SHF specification appeared to occur normally as judged by *latent TGF β binding protein 3* (*ltbp3*) expression [63]. However, combined inhibitions of *nkx2.5* and *nkx2.7* caused an increase in atrial tissue formation at the apparent expense of the ventricle [64] and opposed the specification of *Isl1+* SHF progenitors [33]. In *Drosophila*, the *NK4* ortholog *tinman* inhibits *Collier* expression during muscle-type diversification [65], suggesting that a negative regulatory interaction between NK4 and COE orthologs is part of an ancient network for muscle specification. Future studies will be required to determine if SHF progenitors can also be re-specified into *COE*-dependent branchiomeric muscle precursors downstream of *Tbx1/10* in *Nkx2.5* mutants. We propose that a conserved cross-antagonism between *NK4* and *Tbx1/10* orthologs acts upstream of *GATAa* and *COE* orthologs for heart versus pharyngeal muscle fate specification within an ancestral lineage of cardio-pharyngeal progenitors in tunicates and vertebrates, where this simple ontogenetic motif underlies heart and head muscles development and evolution. More specifically, it is tantalizing to speculate that prolonged Tbx1 expression in the amniote second heart field has fostered the emergence of novel heart compartments by antagonizing heart differentiation, thus maintaining a proliferative progenitor state in the SHF and expanding on the ancestral heterochrony between first and second heart fields' specification that is observed in extant chordates.

## Materials and Methods

### Embryo Preparation

Gravid *Ciona intestinalis* adults were purchased from M-Rep (San Diego, CA). Isolation of gametes, fertilization, dechorionation, electroporation, and development were conducted as described [66]–[69]. Juveniles were obtained by letting the larvae settle and metamorphose on plastic Petri dishes. Filtered artificial sea water was supplemented with antibiotics (streptomycin and penicillin, 50 µg/mL each) and changed daily. The amount of DNA for electroporation was typically 70 µg, except for Mesp>nls:lacZ (50 µg) and for Mesp>lifeact:mCherry (20 µg, Lifeact fusions appeared toxic at higher concentrations). Embryos were fixed at the different developmental stages for 2 h in 4% MEM-PFA and stored in 75% ethanol at −20°C [66].

### Molecular Cloning and Sequence Analyses

The coding sequences (CDS) of *NK4* and *Tbx1/10* (Genbank Accession: KC196542) were amplified by RT-PCR from total RNA isolated from 16 hpf *Ciona intestinalis* larvae using the following primers: NK4_CDS_F, 5′-AAAGGGCCCAAACCATGATTCCTAGTCCGGTTGGATCGACT-3′; NK4_CDS_R, 5′-TTGCTCAGCTCACGTGCACAGCCCAAGCTTAT-3′. The CDS were cloned downstream of the *FoxF* minimal TVC enhancer (FoxF-TVC) fused to the basal promoter of the *Friend of GATA* gene (bpFOG) [70]. The Tbx1/10 coding sequence was amplified using Tbx1 CDS NotI F2, 5'-AAAGCGGCCGCAACCATGTCTGCCCAAATTGCAGTCGGTCACCAT, and Tbx1 CDS BlpI R2, AATGCTCAGCTGACAAGAAACGCTCTCATCTGC.

The N448K point mutation of *NK4* was introduced by overlapping PCR strategy using the primers NK4_N448K _F, 5′-AAGATCTGGTTCCAAAAGCGTCGATACAAATGTAAACGAATGCGACAAGA-3′ and NK4_N448K_R, ACATTTGTATCGACGCTTTTGGAACCAGATCTTGACCTGGGTGGAAGT.

mCherry was fused to N-terminal LifeAct peptide [71] using double-stranded DNA oligonucleotides and subcloned downstream of the *Mesp* enhancer (Mesp>lifeact:mCherry) to visualize the cell cortices of B7.5 lineage cells. Mesp>nls:lacZ was used as described previously [16].

### FISH-IHC

Double FISH-IHC was performed following a protocol modified from Christiaen et al. [66]. Hybridized probes were revealed using the Tyramide Signal Amplification (TSA) with either Cyanine5 or Fluorescein TSA Plus Evaluation Kits (Perkin Elmer, MA). Anti-Digoxigenin-POD Fab fragment (Roche, IN) was first used to detect Digoxigenin-conjugated probe. Anti–β-galactosidase monoclonal mouse antibody (Promega) was co-incubated with anti-DIG-POD for immunodetection of Mesp>nls:lacZ products. A 10 min treatment with WB2 buffer (50% formamide, 2× SSC, 0.1% Tween20) at 55°C was used to denature anti-Digoxigenin-POD antibody after the first Fluorescein TSA reaction [72]. Anti-Fluorescein-POD Fab fragment (Roche, IN) was added to detect the fluorescein-conjugated probe following and revealed by Cyanine5 TSA reaction. Goat anti-mouse secondary antibodies coupled with AlexaFluor-555 were used following the second TSA reaction to detect β-galactosidase-bound mouse antibodies.

### Confocal Imaging and Heart Volume Quantification

Samples were mounted in 50% glycerol/PBS with 2% DABCO. Images were acquired with a Leica TCS SP5 or a Leica TCS SP8 X confocal microscope, using 20× or 63× objectives. Z-stacks were acquired 1.5 µm (20×) or 1 µm (63×) z steps. Maximum projections were processed with maximum projection tools from the LEICA software LAS-AF.

The measurements of juvenile heart volume were conducted using Volocity 5 (PerkinElmer, MA). Volume of Mesp>nls:lacZ labeled heart cells was detected on confocal stacks. Three filters were set for objective detection as following: (1) regions of interest were segmented by percentage intensity (lower threshold, 20%; upper threshold, 100%), (2) noise was removed from objects with a Fine Filter setting, And (3) touching objects separated with object size guide as 100 µm^3^. Quantifications were exported into Excel for further analysis.

### shRNA-Mediated RNAi Knock-Down of Tbx1/10 Function

The coding sequence of Ci-Tbx1/10 was used to identify siRNA target sites with the DSIR [73] and Public TRC Portal (http://www.broadinstitute.org/rnai/public/seq/search) algorithms. Candidate 21-mer target sites were trimmed to 20 mer, and those that begin with A or G and end with A were further considered. Candidate 20 mer were BLASTed against the *Ciona intestinalis* genome on the Ghost database and selected to avoid polymorphic region as well as 20 mer with potential off-targets (i.e., >16 nucleotide between positions 2 and 17 on the antisense). Target sense sequences were reverse-complemented, and sense and antisense sequences were combined to mimick the hairpin structure corresponding to the microRNA miR-2213 [38]. A vector containing the *Ciona U6* promoter [36] was used to clone complementary oligos encoding four shRNA constructs: shTbx1-A-F, GCACAAGTccACttAGATTcTGTAATTaGtAACATAATCTGGGTAAACTTGTGttttt; shTbx1-A-R, aattaaaaaCACAAGTTTACCCAGATTATGTTaCtAATTACAgAATCTaaGTggACTTGTGC; shTbx1-B-F, gCTGGTtAaaCAttGATATcTGTAATTaGtAACATATATCGGTGGGTGACCAGTtttt; shTbx1-B-R, aattaaaaACTGGTCACCCACCGATATATGTTaCtAATTACAgATATCaaTGttTaACCAGc; shTbx1-C-F, gGTtGGTCcaCATAGTTATcTGTAATTaGtAACATATAACTATGGTGACCGACTtttt; shTbx1-C-R, aattaaaaAGTCGGTCACCATAGTTATATGTTaCtAATTACAgATAACTATGtgGACCaACc; shTbx1-D-F:, gTTCACTgaaAGtAtGATAcTGTAATTaGtAACATTATCGTGCTGGTAGTGAATtttt; and shTbx1-D-R, aattaaaaATTCACTACCAGCACGATAATGTTaCtAATTACAgTATCaTaCTttcAGTGAAc. Sensor assays using a GFP:Tbx1/10 fusion indicated that shRNA constructs A, B and A, D were the most efficient pairwise combinations (unpublished data). The data showed used 30 µg each of U6>shTbx1/10-A and -B per electroporation.

### Chromatin Immunoprecipitation (ChIP)

A 2xFLAG tag was cloned to the C terminus of coding sequences to make the following constructs: FoxF>GFP:2xFLAG, FoxF>dnNK4:2xFLAG, FoxF>NK4:2xFLAG. We used 50 µg of plasmid for each electroporation. The larvae from three electroporations were combined as one ChIP sample. All samples were harvested at 16 hpf and cross-linked at 22°C for 15 min by adding formaldehyde to the final concentration of 1%. Chromatin extracted from larvae was sheared into 400 bp fragments by S220 Focused-ultrasonicator (Covaris, MA). The cross-linked enhancer fragments were incubated with DYKDDDDK tag antibody (Cat. No: 2368, Cell Signaling Technology, MA) overnight and then captured by Dynabeads M-280 sheep anti-Rabbit IgG (Invitrogen Dynal AS, Oslo, Norway). Then, enriched DNA were treated by RNaseA and proteinase K and purified using NucleoSpin Gel and PCR Clean-up kit (Machery-Nagel, Düren, Germany).

### Quantitative PCR (qPCR)

Each ChIP sample was obtained in three independent biological replicates. Two pairs of primers were designed within the minimal *COE* enhancer. Two pairs of primers were also used to amplify the enhancer region of *Ciona Brachyury* [44] and provide an off-target negative/loading control for qPCR. The primers used in qPCR are listed as below: En_CiCOE_F1 5′ GTCGCTGAGGAAGTCATCATT 3′; En_CiCOE_R1 5′ GAACCAATAAGGACGGGTGAA 3′; En_CiCOE_F2 5′ TGGCGGCTGGCTAATTAAA 3′; En_CiCOE_R2 5′ TTCCCTGCAGACAAATAATGGA 3′; En_CiBra_F1 5′ GGCGCACTTTCAACAAACA 3′; En_CiBra_R1 5′ TCTGCCTCCAAATCACACTC 3′; En_CiBra_F2 5′ CACGCAAGACAATGGGAAAG 3′; En_CiBra_R2 5′ GGTGGCGCTCTATGTTTACT 3′. Quantitative PCR was performed using the SYBR Green method on a LightCycler 480 system (Roche Diagnostics, IN). Each qPCR was performed in technical triplicates, and the average values were used for subsequent calculations. Fold enrichments were calculated using the ΔΔCt method: For each ChIP sample and corresponding input, the ΔCt for *En_COE_1* and *En_COE_2* versus the average *Brachyury* was calculated first. Then ChIP versus input ΔΔCt was calculated for each *En_CiCOE* primer pair and each sample. Finally, fold enrichment over mock control was calculated for each replicate. Average fold enrichments relative to mock controls are showed in Figure 6. Similar values were obtained relatively to the GFP:2xFLAG negative control (Table S2).

## Supporting Information

Figure S1 **Conserved features of** ***NKX2-5*** **homedomain proteins across species.** (A) Alignment of *NK4* orthologues across species using ClustalX 2.1 (http://www.clustal.org/clustal2/). *CiNK4*, *Ciona intestinalis*, NP_001071957, 623 aa [25],[77]; *CsNKX2-5*, *Ciona savignyi*, BAA25399, 595aa; *GgNKX2-5*, *Gallus gallus*, NP_990495, 294 aa [78]; *XlNKX2-5*, *Xenopus laevis*, NP_001080190, 299 aa [79]; *MmNKX2-5*, *Mus musculus*, NP_032726, 318 aa [80]; *HsNKX2-5*, *Homo sapiens*, NP_004378, 324 aa [81]; *DrNKX2-5*, *Danio rerio*, NP_571496, 314 aa [82]; *SkNKX2-3/5*, *Saccoglossus kowalevskii*, NP_001158401, 268 aa[83]; *Tinman*, *Drosophila melanogaster*, NP_524433, 416 aa [84]. Tinman domain, Homeobox, NK2-SD domain, and Tyrosine-Rich Domain [85] are highlighted in boxes. *CiNK4* lacks the Nkx2-5 box and GIRAW motif. (B) Domain structure of *CiNK4*. Asparagine (N) at amino acid residue position 448, which is critical for DNA binding capability of the homeobox [28], is mutated to Lysine (K) to generate the dominant negative version of NK4 (dnNK4).
(TIF)

Figure S2 **Endogenous expressions of** ***NK4*** **.** (A–D) *NK4* endogenous expression pattern during TVC lineage specification. Mesp>LifeAct:mCherry (blue) and Mesp>nls:lacZ (red) were detected by immunostaining, indicating cell boundary and nucleus, respectively; *NK4* mRNAs (green) were detected by FISH. *NK4* transcripts are present throughout the TVC lineage and endoderm from 12 to 20 hpf at 18°C. Scale bar, 25 µm. (A–D) FISH detection of *NK4* transcripts (green) at 48 hpf (E), 72 hpf (F). Red, Mesp>nls:lacZ positive B7.5 lineage cells; the heart areas (dotted line square) were rescanned at higher magnification (63× objective; E′, F′). *NK4* transcripts were not detected in the juvenile heart. Scale bar, 10 µm.
(TIF)

Figure S3 ***COE*** **expression initiates prior to that of** ***Islet*** **.** (A) Proportions of embryos and larva showing *COE* (blue) or *Islet* (red) expression at indicated stages of TVC development (the diagrams correspond to the stages described in Figure 1A). *COE* expression starts in the ASM precursors immediately after the second asymmetric division. *Islet* expression starts after *COE*, throughout the lineage first, and quickly becomes stronger in the ASMs. (B) Initiation of *COE* expression visualized in early ASMs by two nuclear dots, indicating that this cDNA probe labels nascent transcripts. Ventral view of larva with TVC lineage progenitors in early class 2 pattern. Larva is electroporated with Mesp>nls:lacZ to mark the TVC lineage (red). DAPI channel to visualize the nucleus (blue). *COE* transcripts are detected by FISH (green). TVC lineage cells (squared) are magnified. (C–E) *Islet* nascent transcripts detected by *Islet* intron-specific probe (green). TVC lineage is marked with Mesp>nls:lacZ (red). *Islet* transcription is activated in all the TVC derivatives around 18 hpf, but exclusively in the ASM precursors (open arrowheads) at 20 hpf. Scale bar, 25 µm.
(TIF)

Figure S4 ***NK4*** **represses** ***COE*** **in TVC derivatives.** (A–C, E–G) FISH of *COE* (green) in 28 hpf (A–C) and 24 hpf (E–G) larvae; FoxF>dnNK4 induces ectopic *COE* expression and results in extra-numerary TVC lineage cells to contribute to the formation of the ASM ring (B). Conversely, FoxF>NK4 represses *COE* and inhibits ASM ring formation (C). Mesp>nls:lacZ (red) labels B7.5 lineage cells; *COE* transcripts (green) detected by FISH. White dotted lines indicate the midline. Scale bar, 25 µm. Counts of *COE*+/− (D, H, I) and *GATAa*+/− (J) cells following manipulations of NK4 activity at 28 hpf (D), 24 hpf (H), and 18 hpf (I, J). Student's *t* tests compare each experimental condition to the control. **p<0.05 and *0.05<*p*<0.1. The significant increase in *GATAa*− cell numbers observed in (J) is due to a slight increase in the total number of cells per half in these larvae, which could be caused by premature divisions in the dnNK4 condition (see text for details). (K) FISH-IHC with *COE* intronic probes at indicated stages of larvae electroporated with indicated constructs. Note that no ectopic *COE* expression was detected before 17–17.5 hpf.
(TIF)

Figure S5 ***NK4*** **regulates heart and ASM specification via** ***COE*** **.** (A–F) Lateral views of 28 hpf larvae, showing the ventral heart precursors and dorso-lateral ASM rings. Mesp>nls:lacZ (red immunostaining) marks B7.5 lineage cells. Larvae hybridized with digoxigenin-conjugated *MHC3* probe (green). (A) In control larvae, four to six of the ASM ring cells express *MHC3*. (B) FoxF>COE:WRPW abolishes ASM migration, ring formation, and *MHC3* expression in TVC derivatives. (C–C′) FoxF>COEΔ321 has mild or no significant effects on second migration, causes ASMs to cluster instead of forming a ring in 31% (23/74) (the cluster phenotype is scored independently) of the larvae (C′), and inhibits *MHC3* expression. (D) FoxF>NK4 increases the number of migrating and *MHC3+* cells. (E) FoxF>COE:WRPW inhibits the effects of FoxF>dnNK4; neither second migration nor *MHC3* expression are observed. (F) FoxF>COEΔ321 also inhibits the effects of FoxF>dnNK4; fewer cells migrates to the siphon placode and fewer *MHC3+* cells are observed in the ASM ring. (G) Numbers of *MHC3+*/− cells per half in indicates conditions. (H) Numbers of cells that migrates to the Atrial Siphon Primordium per half. Student's *t* tests compare each experimental condition to the control. **p<0.05.
(TIF)

Figure S6 ***NK4*** **represses** ***Islet*** **in TVC lineage cells.** FISH of *Islet* in 20 (A–C), 24 (D–F), and 28 hpf (G–I) larvae. In control larvae, *Islet* is expressed at higher levels in ASM precursors than in heart precursors. Targeted expression of dnNK4 causes up-regulation of *Islet* in heart precursors, while overexpression of *NK4* inhibits *Islet* in all TVC derivatives. Asterisk, *Islet* expression in endodermal cells; dotted line, ventral midline. Scale bar, 25 µm.
(TIF)

Figure S7 **Endogenous expression pattern of** ***GATAa*** **during TVC lineage specification.** *GATAa* mature (green) (A–D) and nascent (E–L) (green) transcripts were detected by FISH. Mesp>nls:LacZ (red) were detected by immunostaining to indicate cell nucleus. ASM precursors, open arrowheads; SHPs, white arrowheads; FHPs, arrows. Dotted lines indicate the midline. Scale bar, 10 µm. *GATAa* transcription is active in the TVCs at stage 23 (corresponding to 14 hpf at 16°C) (A, E) and then stopped after the first asymmetric division (B, F, G). The transcription reactivates at 16 hpf in FHP (H–L).
(TIF)

Figure S8 **Mapping a minimal ASM enhancer for** ***COE*** **.** (A) Snapshots of the ANISEED and VISTA browsers showing the KH2008 transcript models for *COE* and sequence conservation between *Ciona intestinalis* and *C. savignyi*. (B) Map and coordinates (relative to the +1 ATG) of the genomic fragments that were cloned upstream of bpFOG>mCherry and tested in reporter gene expression assays. Percentages indicate the percentage of 21 hpf larvae showing ASM-specific mCherry expression among Mesp>GFP+ larvae. These constructs mapped a minimal ASM enhancer encompassing a conserved noncoding sequence ∼2.5 kb upstream of *COE*. (C) Example of a 21 hpf larva showing mCherry expression in the ASM (arrow) but not in the heart (plain arrowhead), where only GFP is detected because Mesp>GFP is active early and marks all the descendants of the B7.5 blastomeres.
(TIF)

Table S1 **Heart volumes in control and experimental juveniles.** Fertilized eggs were electroporated with Mesp>nls:lacZ and indicated constructs driven by FoxF(TVC)bpFOG. Electroporated larvae were raised until 72 hpf juvenile stage, fixed, and stained for *MHC2*, *MHC3*, and β-galactosidase expression using FISH-IHC. The total volume of βgal+ nuclei per half was measured using Volocity (see the Materials and Methods section for details). Volumes are expressed in µm^3^.
(DOCX)

Table S2 **Relative qPCR values for ChIP samples.** Values were obtained as described in the Materials and Methods section. Fold enrichment relative to either the “mock” of “GFP” negative controls are shown. “Folds” indicate enrichments of COE enhancers in the NK4:2xFLAG samples relative to the other samples. The *p* values are for indicated pair-wise comparisons and were performed in Excel using the TTEST function with two-tail distributions for two-sample of unequal variance.
(DOCX)

## Abbreviations

ASM: atrial siphon muscle
ATM: anterior tail muscle
*COE*: *Collier/Olf/EBF*
COEΔ321: dominant negative form of COE lacking the N-terminal DNA binding domain
DGS/CVFS: DiGeorge/Cardio-Velo-Facial syndrome
dnNK4: dominant-negative N448K DNA-binding domain mutant of NK4
FHF: first heart field
FHP: first heart precursor
LoM: longitudinal muscle
*MHC3*: *myosin heavy chain 2*
*MHC3*: *myosin heavy chain 3*
RNAi: RNA interference
SHF: second heart field
SHP: second heart precursor
shRNA: short hairpin RNA
TVC: trunk ventral cell
