# Supplementary figures and images for: NK4 Antagonizes Tbx1/10 to Promote Cardiac versus Pharyngeal Muscle Fate in the Ascidian Second Heart Field
Source: PLoS Biol. 2013 Dec 3;11(12):e1001725. doi: 10.1371/journal.pbio.1001725 (PMC3849182; doi:10.1371/journal.pbio.1001725)

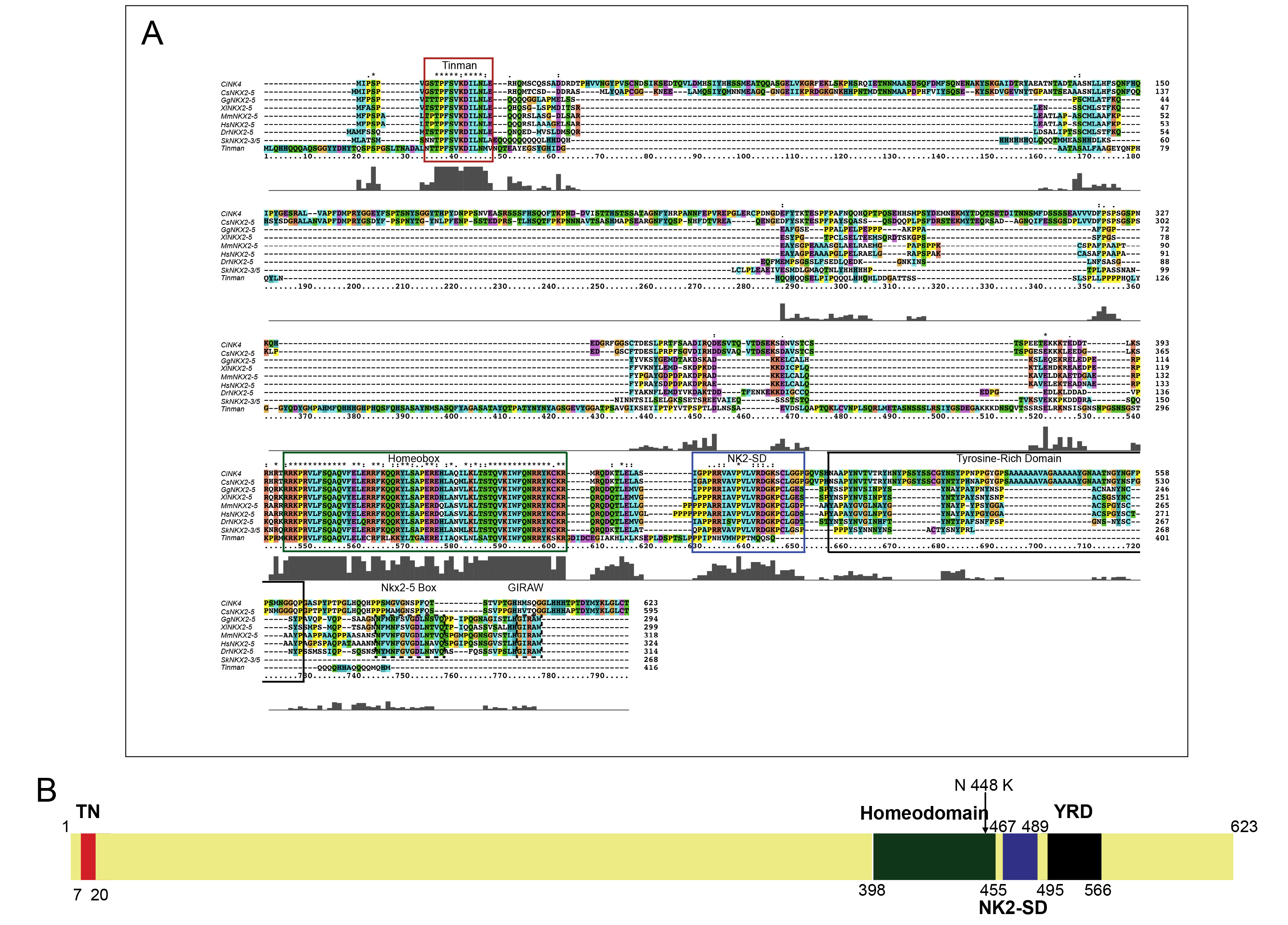

Supplement: Figure S1 — Conserved features of NKX2-5 homedomain proteins across species. (A) Alignment of NK4 orthologues across species using ClustalX 2.1 (http://www.clustal.org/clustal2/). CiNK4, Ciona intestinalis, NP_001071957, 623 aa [25],[77]; CsNKX2-5, Ciona savignyi, BAA25399, 595aa; GgNKX2-5, Gallus gallus, NP_990495, 294 aa [78]; XlNKX2-5, Xenopus laevis, NP_001080190, 299 aa [79]; MmNKX2-5, Mus musculus, NP_032726, 318 aa [80]; HsNKX2-5, Homo sapiens, NP_004378, 324 aa [81]; DrNKX2-5, Danio rerio, NP_571496, 314 aa [82]; SkNKX2-3/5, Saccoglossus kowalevskii, NP_001158401, 268 aa[83]; Tinman, Drosophila melanogaster, NP_524433, 416 aa [84]. Tinman domain, Homeobox, NK2-SD domain, and Tyrosine-Rich Domain [85] are highlighted in boxes. CiNK4 lacks the Nkx2-5 box and GIRAW motif. (B) Domain structure of CiNK4. Asparagine (N) at amino acid residue position 448, which is critical for DNA binding capability of the homeobox [28], is mutated to Lysine (K) to generate the dominant negative version of NK4 (dnNK4). (TIF) [file pbio.1001725.s001.tif]

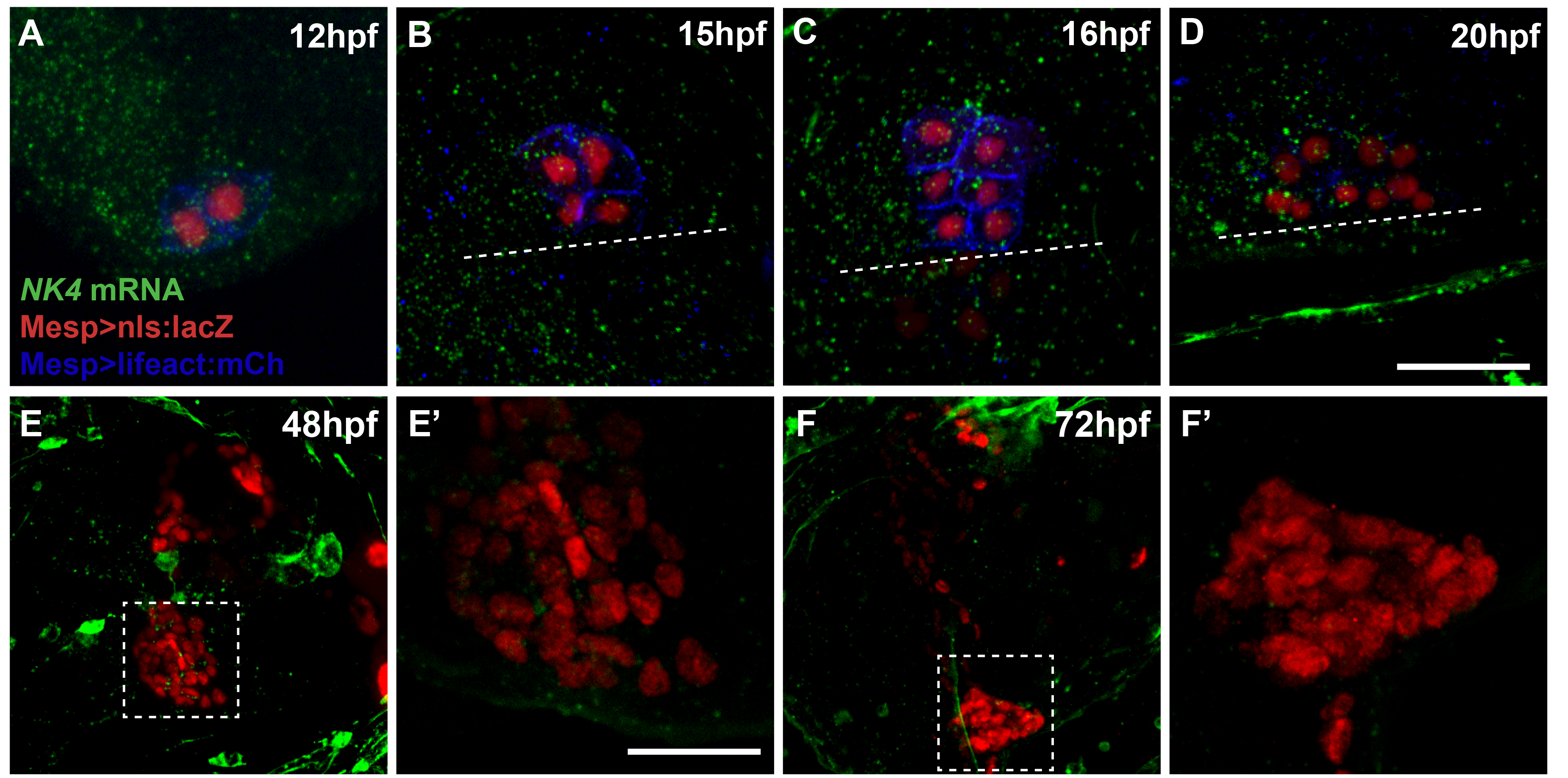

Supplement: Figure S2 — Endogenous expressions of NK4 . (A–D) NK4 endogenous expression pattern during TVC lineage specification. Mesp>LifeAct:mCherry (blue) and Mesp>nls:lacZ (red) were detected by immunostaining, indicating cell boundary and nucleus, respectively; NK4 mRNAs (green) were detected by FISH. NK4 transcripts are present throughout the TVC lineage and endoderm from 12 to 20 hpf at 18°C. Scale bar, 25 µm. (A–D) FISH detection of NK4 transcripts (green) at 48 hpf (E), 72 hpf (F). Red, Mesp>nls:lacZ positive B7.5 lineage cells; the heart areas (dotted line square) were rescanned at higher magnification (63× objective; E′, F′). NK4 transcripts were not detected in the juvenile heart. Scale bar, 10 µm. (TIF) [file pbio.1001725.s002.tif]

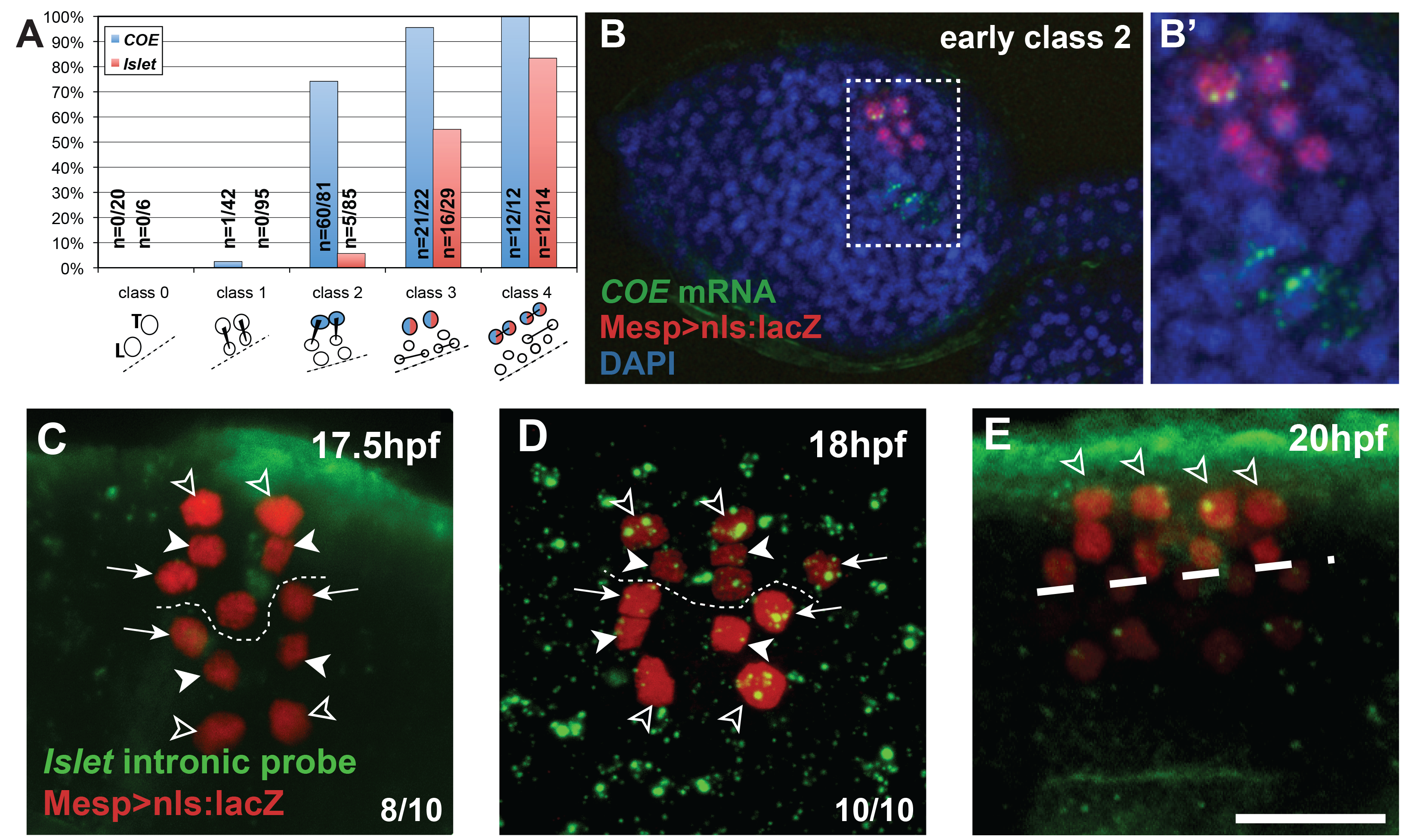

Supplement: Figure S3 — COE expression initiates prior to that of Islet . (A) Proportions of embryos and larva showing COE (blue) or Islet (red) expression at indicated stages of TVC development (the diagrams correspond to the stages described in Figure 1A). COE expression starts in the ASM precursors immediately after the second asymmetric division. Islet expression starts after COE, throughout the lineage first, and quickly becomes stronger in the ASMs. (B) Initiation of COE expression visualized in early ASMs by two nuclear dots, indicating that this cDNA probe labels nascent transcripts. Ventral view of larva with TVC lineage progenitors in early class 2 pattern. Larva is electroporated with Mesp>nls:lacZ to mark the TVC lineage (red). DAPI channel to visualize the nucleus (blue). COE transcripts are detected by FISH (green). TVC lineage cells (squared) are magnified. (C–E) Islet nascent transcripts detected by Islet intron-specific probe (green). TVC lineage is marked with Mesp>nls:lacZ (red). Islet transcription is activated in all the TVC derivatives around 18 hpf, but exclusively in the ASM precursors (open arrowheads) at 20 hpf. Scale bar, 25 µm. (TIF) [file pbio.1001725.s003.tif]

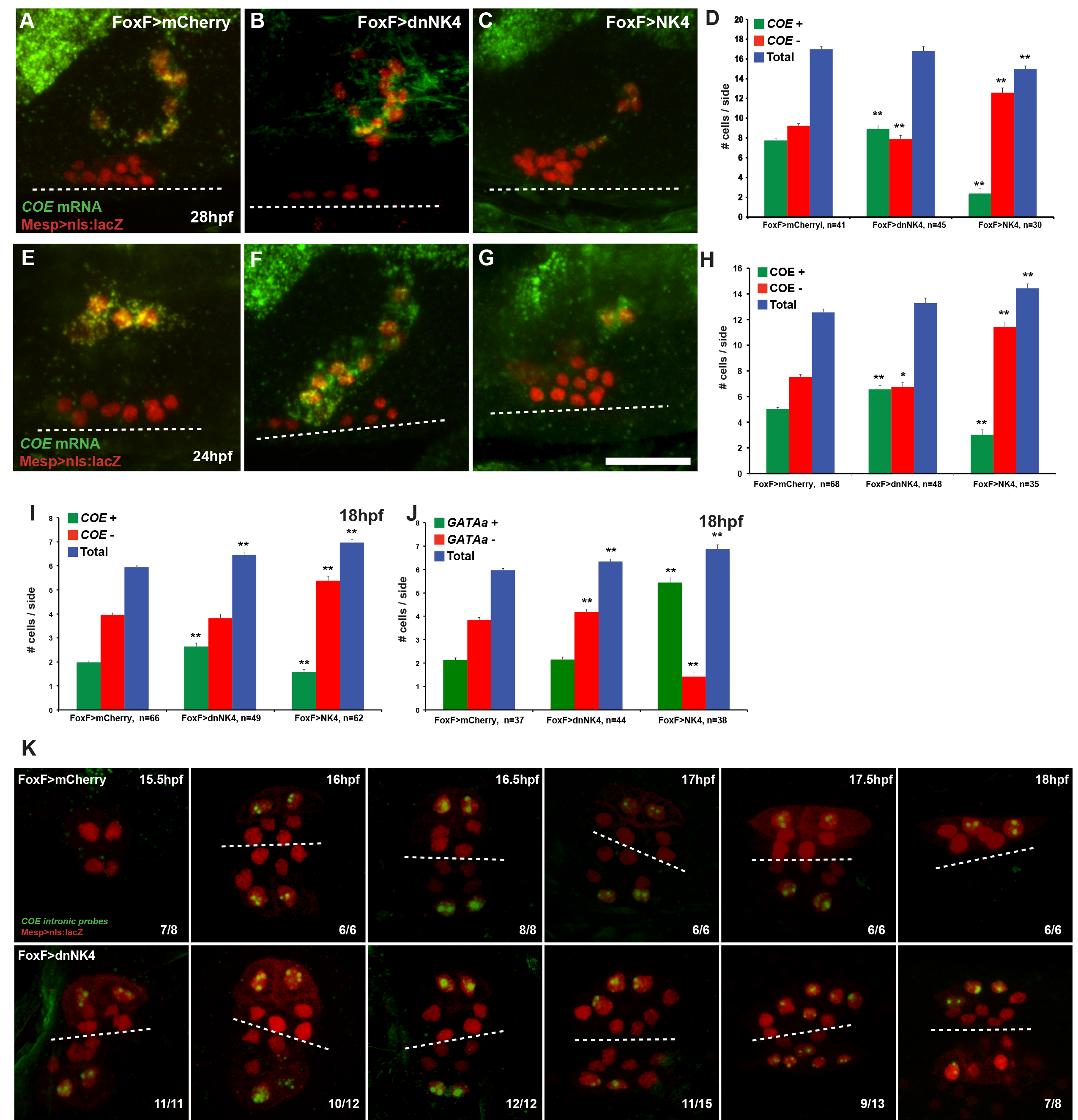

Supplement: Figure S4 — NK4 represses COE in TVC derivatives. (A–C, E–G) FISH of COE (green) in 28 hpf (A–C) and 24 hpf (E–G) larvae; FoxF>dnNK4 induces ectopic COE expression and results in extra-numerary TVC lineage cells to contribute to the formation of the ASM ring (B). Conversely, FoxF>NK4 represses COE and inhibits ASM ring formation (C). Mesp>nls:lacZ (red) labels B7.5 lineage cells; COE transcripts (green) detected by FISH. White dotted lines indicate the midline. Scale bar, 25 µm. Counts of COE+/− (D, H, I) and GATAa+/− (J) cells following manipulations of NK4 activity at 28 hpf (D), 24 hpf (H), and 18 hpf (I, J). Student's t tests compare each experimental condition to the control. **p<0.05 and *0.05<p<0.1. The significant increase in GATAa− cell numbers observed in (J) is due to a slight increase in the total number of cells per half in these larvae, which could be caused by premature divisions in the dnNK4 condition (see text for details). (K) FISH-IHC with COE intronic probes at indicated stages of larvae electroporated with indicated constructs. Note that no ectopic COE expression was detected before 17–17.5 hpf. (TIF) [file pbio.1001725.s004.tif]

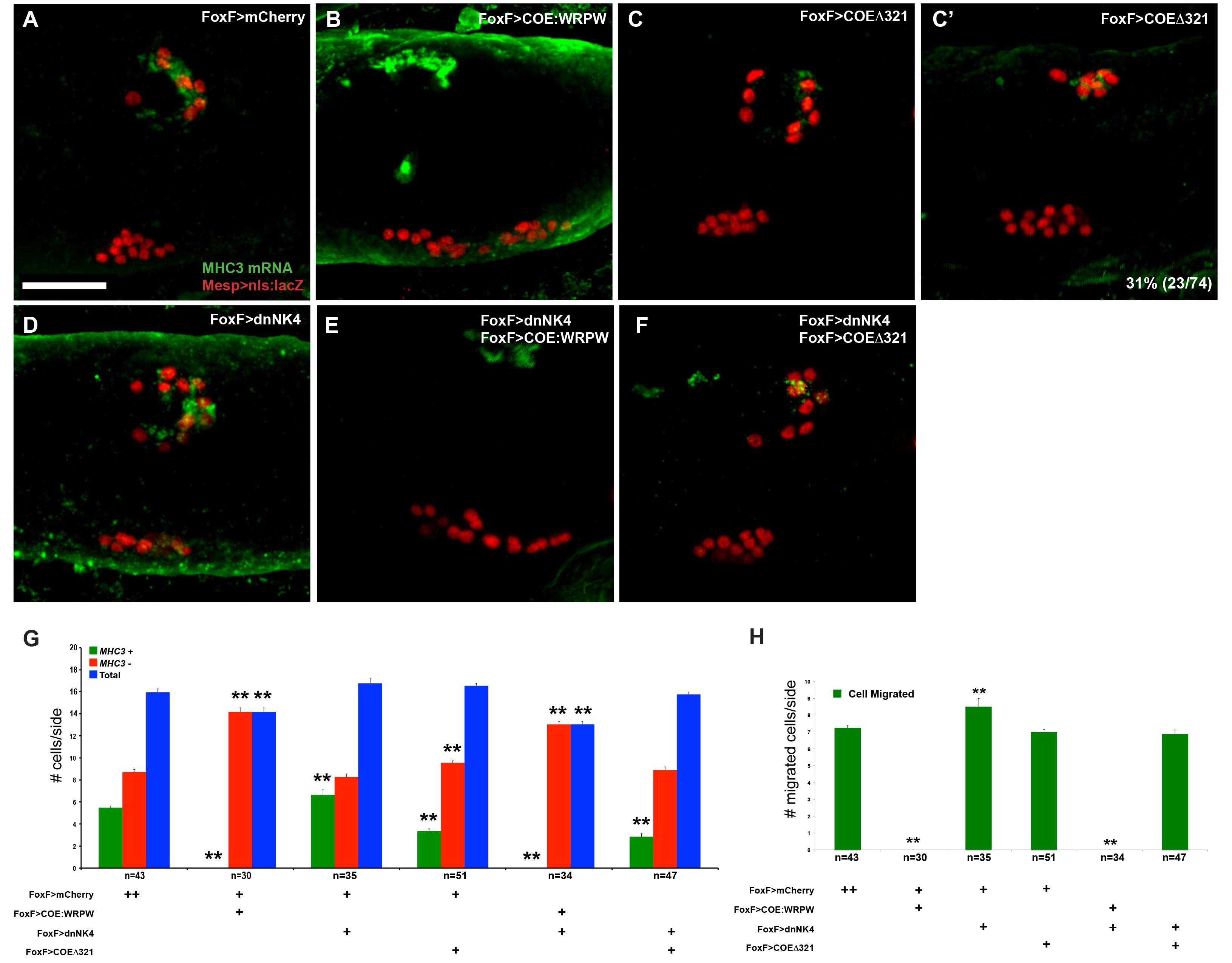

Supplement: Figure S5 — NK4 regulates heart and ASM specification via COE . (A–F) Lateral views of 28 hpf larvae, showing the ventral heart precursors and dorso-lateral ASM rings. Mesp>nls:lacZ (red immunostaining) marks B7.5 lineage cells. Larvae hybridized with digoxigenin-conjugated MHC3 probe (green). (A) In control larvae, four to six of the ASM ring cells express MHC3. (B) FoxF>COE:WRPW abolishes ASM migration, ring formation, and MHC3 expression in TVC derivatives. (C–C′) FoxF>COEΔ321 has mild or no significant effects on second migration, causes ASMs to cluster instead of forming a ring in 31% (23/74) (the cluster phenotype is scored independently) of the larvae (C′), and inhibits MHC3 expression. (D) FoxF>NK4 increases the number of migrating and MHC3+ cells. (E) FoxF>COE:WRPW inhibits the effects of FoxF>dnNK4; neither second migration nor MHC3 expression are observed. (F) FoxF>COEΔ321 also inhibits the effects of FoxF>dnNK4; fewer cells migrates to the siphon placode and fewer MHC3+ cells are observed in the ASM ring. (G) Numbers of MHC3+/− cells per half in indicates conditions. (H) Numbers of cells that migrates to the Atrial Siphon Primordium per half. Student's t tests compare each experimental condition to the control. **p<0.05. (TIF) [file pbio.1001725.s005.tif]

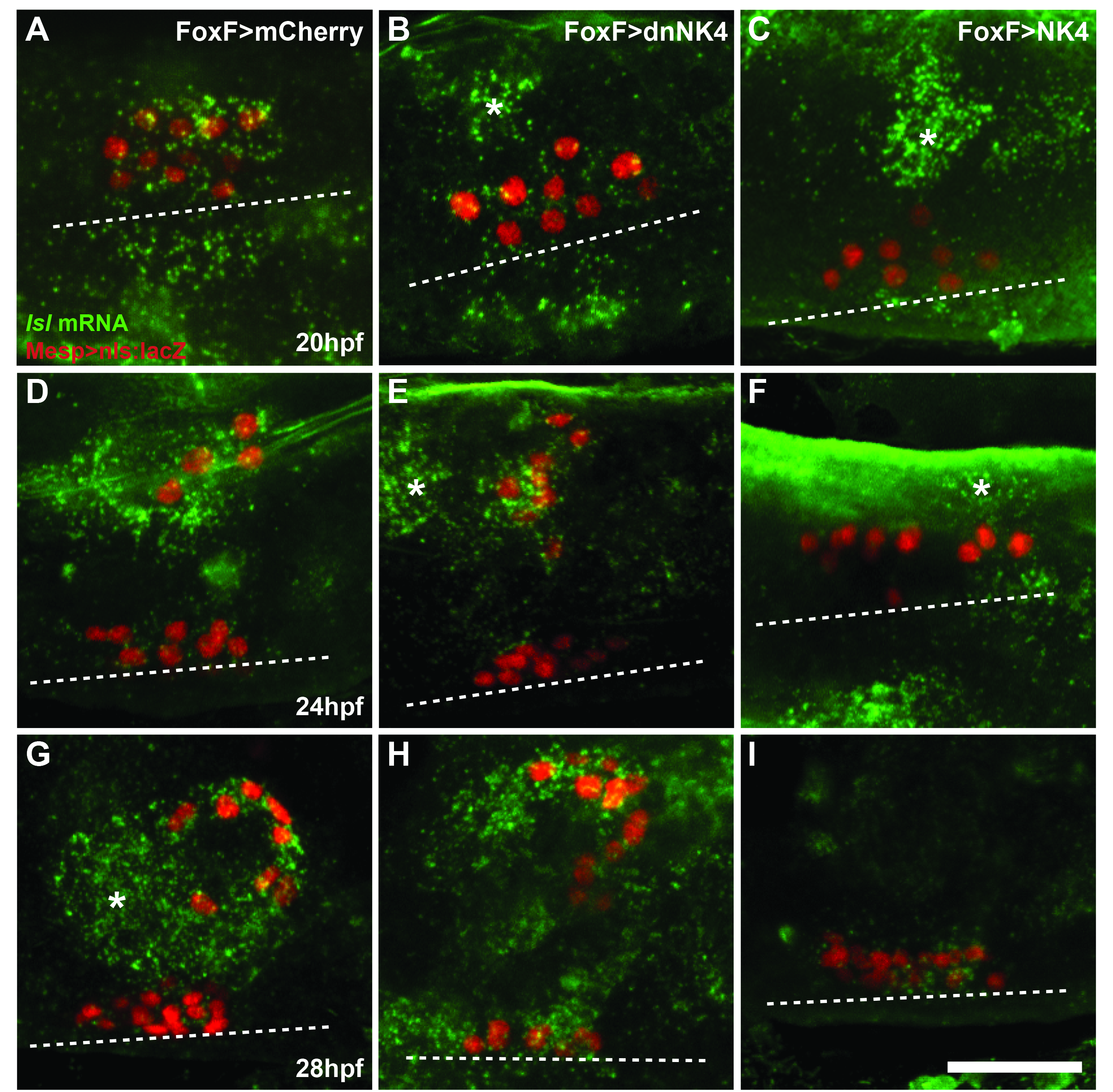

Supplement: Figure S6 — NK4 represses Islet in TVC lineage cells. FISH of Islet in 20 (A–C), 24 (D–F), and 28 hpf (G–I) larvae. In control larvae, Islet is expressed at higher levels in ASM precursors than in heart precursors. Targeted expression of dnNK4 causes up-regulation of Islet in heart precursors, while overexpression of NK4 inhibits Islet in all TVC derivatives. Asterisk, Islet expression in endodermal cells; dotted line, ventral midline. Scale bar, 25 µm. (TIF) [file pbio.1001725.s006.tif]

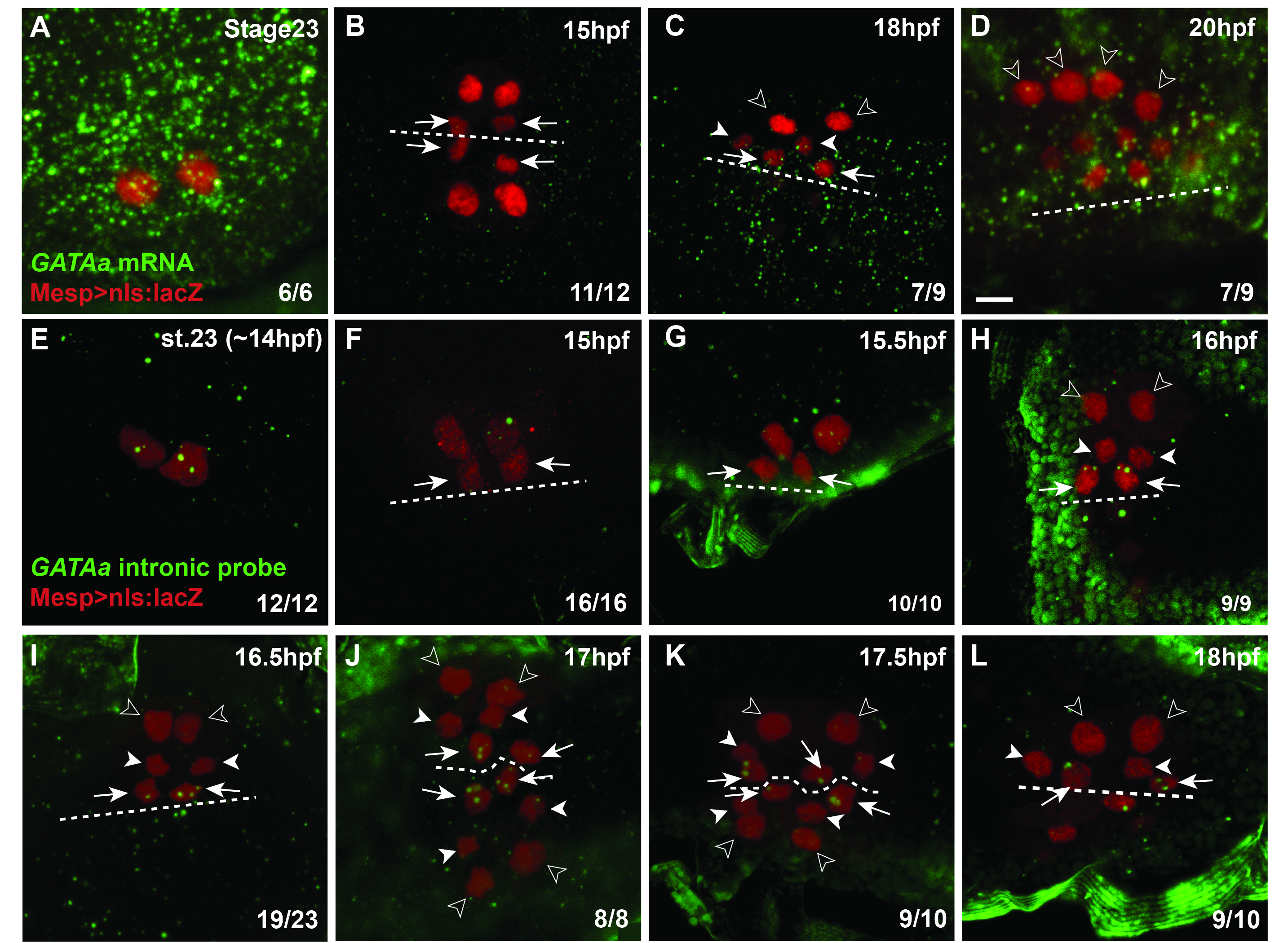

Supplement: Figure S7 — Endogenous expression pattern of GATAa during TVC lineage specification. GATAa mature (green) (A–D) and nascent (E–L) (green) transcripts were detected by FISH. Mesp>nls:LacZ (red) were detected by immunostaining to indicate cell nucleus. ASM precursors, open arrowheads; SHPs, white arrowheads; FHPs, arrows. Dotted lines indicate the midline. Scale bar, 10 µm. GATAa transcription is active in the TVCs at stage 23 (corresponding to 14 hpf at 16°C) (A, E) and then stopped after the first asymmetric division (B, F, G). The transcription reactivates at 16 hpf in FHP (H–L). (TIF) [file pbio.1001725.s007.tif]

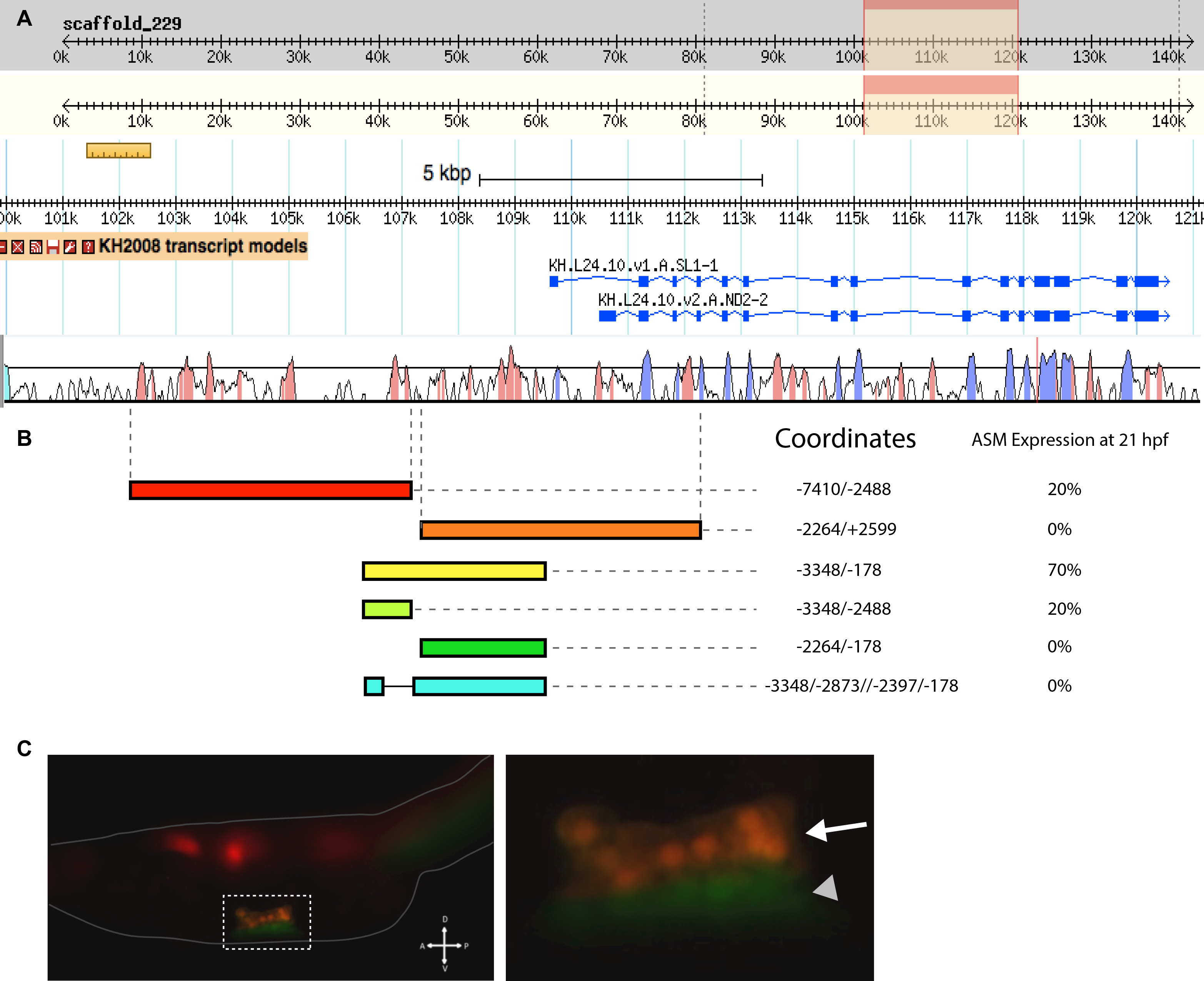

Supplement: Figure S8 — Mapping a minimal ASM enhancer for COE . (A) Snapshots of the ANISEED and VISTA browsers showing the KH2008 transcript models for COE and sequence conservation between Ciona intestinalis and C. savignyi. (B) Map and coordinates (relative to the +1 ATG) of the genomic fragments that were cloned upstream of bpFOG>mCherry and tested in reporter gene expression assays. Percentages indicate the percentage of 21 hpf larvae showing ASM-specific mCherry expression among Mesp>GFP+ larvae. These constructs mapped a minimal ASM enhancer encompassing a conserved noncoding sequence ∼2.5 kb upstream of COE. (C) Example of a 21 hpf larva showing mCherry expression in the ASM (arrow) but not in the heart (plain arrowhead), where only GFP is detected because Mesp>GFP is active early and marks all the descendants of the B7.5 blastomeres. (TIF) [file pbio.1001725.s008.tif]
