# Supplementary material for: NK4 Antagonizes Tbx1/10 to Promote Cardiac versus Pharyngeal Muscle Fate in the Ascidian Second Heart Field
Source: PLoS Biol. 2013 Dec 3;11(12):e1001725. doi: 10.1371/journal.pbio.1001725 (PMC3849182; doi:10.1371/journal.pbio.1001725)
Supplement: Table S1 — Heart volumes in control and experimental juveniles. Fertilized eggs were electroporated with Mesp>nls:lacZ and indicated constructs driven by FoxF(TVC)bpFOG. Electroporated larvae were raised until 72 hpf juvenile stage, fixed, and stained for MHC2, MHC3, and β-galactosidase expression using FISH-IHC. The total volume of βgal+ nuclei per half was measured using Volocity (see the Materials and Methods section for details). Volumes are expressed in µm3. (DOCX) [file pbio.1001725.s009.docx]

| #animal | mCherry | dnNK4 | NK4 | Tbx1 | Tbx1/dnNK4 | Tbx1/NK4 |
| --- | --- | --- | --- | --- | --- | --- |
| 1 | 1624 | 1039 | 5445 | 1332 | 532 | 912 |
| 2 | 1136 | 989 | 4346 | 956 | 0 | 1854 |
| 3 | 1472 | 431 | 3824 | 685 | 0 | 2411 |
| 4 | 2157 | 944 | 4416 | 1019 | 1660 | 2082 |
| 5 | 1390 | 1077 | 5151 | 1371 | 776 | 1682 |
| 6 | 1854 | 984 | 3332 | 1416 | 563 | 2214 |
| 7 | 2161 | 699 | 4734 | 1804 | 984 | 3479 |
| 8 | 2608 | 615 |  | 583 | 567 | 961 |
| 9 |  | 1352 |  | 1016 | 251 | 1980 |
| 10 |  |  |  |  | 625 |  |
| 11 |  |  |  |  | 826 |  |
| 12 |  |  |  |  | 394 |  |
|  |  |  |  |  |  |  |
| AVERAGE | 1800 | 903 | 4464 | 1131 | 598 | 1953 |
| STDEV | 487 | 277 | 732 | 385 | 452 | 772 |
| SEM | 172 | 92 | 277 | 128 | 130 | 257 |
| change/control | 100% | 50% | 248% | 63% | 33% | 108% |
