# Supplementary material for: NK4 Antagonizes Tbx1/10 to Promote Cardiac versus Pharyngeal Muscle Fate in the Ascidian Second Heart Field
Source: PLoS Biol. 2013 Dec 3;11(12):e1001725. doi: 10.1371/journal.pbio.1001725 (PMC3849182; doi:10.1371/journal.pbio.1001725)
Supplement: Table S2 — Relative qPCR values for ChIP samples. Values were obtained as described in the Materials and Methods section. Fold enrichment relative to either the “mock” of “GFP” negative controls are shown. “Folds” indicate enrichments of COE enhancers in the NK4:2xFLAG samples relative to the other samples. The p values are for indicated pair-wise comparisons and were performed in Excel using the TTEST function with two-tail distributions for two-sample of unequal variance. (DOCX) [file pbio.1001725.s010.docx]

| **Reference sample** | **replicate** | **enhancer** | **NK4_mock** | **GFP** | **NK4** | **dnNK4** |
| --- | --- | --- | --- | --- | --- | --- |
| relative to mock (NK4) | #1 | COE_1 | 1.00000 | 0.68302 | 2.88786 | 0.69176 |
|  | #1 | COE_2 | 1.00000 | 0.49655 | 2.32409 | 0.59805 |
|  | #2 | COE_1 | 1.00000 | 0.78007 | 0.94497 | 0.29085 |
|  | #2 | COE_2 | 1.00000 | 0.98737 | 1.13419 | 0.27200 |
|  | #3 | COE_1 | 1.00000 | 0.91489 | 3.74076 | 0.26304 |
|  | #3 | COE_2 | 1.00000 | 0.72615 | 2.89454 | 0.24942 |
|  |  |  |  |  |  |  |
|  |  | AVG | 1.00000 | 0.76467 | 2.32107 | 0.39418 |
|  |  | SEM | 0.00000 | 0.07128 | 0.44602 | 0.08039 |
|  |  |  |  |  |  |  |
|  |  | Folds | 2.32107 | 3.03537 | NA | 5.88828 |
|  |  |  |  |  |  |  |
|  |  | p(NK4-GFP) | 0.01692 |  |  |  |
|  |  | p(NK4-dnNK4) | 0.00702 |  |  |  |
|  |  |  |  |  |  |  |
| relative to GFP | #1 | COE_1 | 1.46409 | 1.00000 | 4.22807 | 1.01279 |
|  | #1 | COE_2 | 2.01391 | 1.00000 | 4.68051 | 1.20442 |
|  | #2 | COE_1 | 1.28194 | 1.00000 | 1.21139 | 0.37285 |
|  | #2 | COE_2 | 1.01279 | 1.00000 | 1.14870 | 0.27548 |
|  | #3 | COE_1 | 1.09303 | 1.00000 | 4.08877 | 0.28751 |
|  | #3 | COE_2 | 1.37713 | 1.00000 | 3.98616 | 0.34349 |
|  |  |  |  |  |  |  |
|  |  | AVG | 1.37382 | 1.00000 | 3.22393 | 0.58275 |
|  |  | SEM | 0.14555 | 0.00000 | 0.65360 | 0.16875 |
|  |  |  |  |  |  |  |
|  |  | Folds | 2.34670 | 3.22393 | NA | 5.53224 |
|  |  |  |  |  |  |  |
|  |  | p(NK4-mock) | 0.03589 |  |  |  |
|  |  | p(NK4-dnNK4 | 0.00882 |  |  |  |
